# Supercritical Carbon Dioxide as a Green Alternative to Achieve Drug Complexation with Cyclodextrins

**DOI:** 10.3390/ph14060562

**Published:** 2021-06-11

**Authors:** Mauro Banchero

**Affiliations:** Department of Applied Science and Technology, Politecnico di Torino, Corso Duca degli Abruzzi, 24, 10129 Torino, Italy; mauro.banchero@polito.it

**Keywords:** cyclodextrin, complexation, supercritical drying, supercritical antisolvent, supercritical impregnation, drug release, bioavailability

## Abstract

Cyclodextrins are widely used in pharmaceutics to enhance the bioavailability of many drugs. Conventional drug/cyclodextrin complexation techniques suffer from many drawbacks, such as a high residual content of toxic solvents in the formulations, the degradation of heat labile drugs and the difficulty in controlling the size and morphology of the product particles. These can be overcome by supercritical fluid technology thanks to the outstanding properties of supercritical CO_2_ (scCO_2_) such as its mild critical point, its tunable solvent power, and the absence of solvent residue after depressurization. In this work the use of scCO_2_ as an unconventional medium to achieve the complexation with native and substituted cyclodextrins of over 50 drugs, which belong to different classes, are reviewed. This can be achieved with different approaches such as the “supercritical solvent impregnation” and “particle-formation” techniques. The different techniques are discussed to point out how they affect the complexation mechanism and efficiency, the physical state of the drug as well as the particle size distribution and morphology, which finally condition the release kinetics and drug bioavailability. When applicable, the results obtained for the same drug with various cyclodextrins, or different complexation techniques are compared with those obtained with conventional approaches.

## 1. Introduction

**Figure 1:**
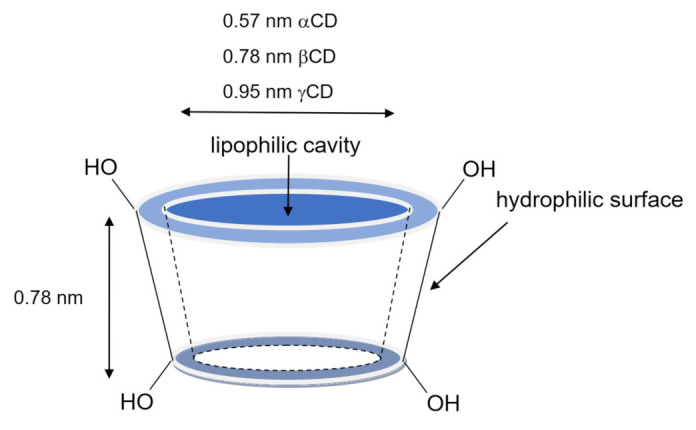
The toroidal shape and dimensions of natural cyclodextrins.

Cyclodextrins are widely used pharmaceutical excipients thanks to their unique properties [1,2]. They are cyclic oligosaccharides composed of different α-D-glucopyranose units, which results in truncated-cone-shaped molecules with a central cavity (Figure 1). The external surface is hydrophilic, while the central cavity is lipophilic and can host suitable-sized guest molecules. The most common naturally occurring cyclodextrins are the α-cyclodextrin (αCD), β-cyclodextrin (βCD) and γ-cyclodextrin (γCD). They differ for the number of glucopyranose units that compose the truncated-cone structure, which can be 6, 7 or 8, and provide different internal lipophilic cavity sizes. In addition to natural cyclodextrins, many chemically modified derivatives are available, which are obtained by substituting some of the hydroxyl groups on the external surface with other functional groups (hydroxypropyl, methyl, triacetyl, etc.) [2,3]. The importance of cyclodextrins in the pharmaceutical field is related to their ability to form inclusion and non-inclusion complexes with a large variety of drugs [1]. This allows favorable changes in the physico-chemical properties of the guest molecule to be achieved, such as increased solubility and bioavailability, controlled dissolution rate, higher stability, and improved organoleptic features [3].

Supercritical fluid technology is employed in several commercial processes [4] and research areas [5,6,7], where supercritical carbon dioxide (scCO_2_) is the most used solvent due to its low critical point (31.2 °C, 7.4 MPa), non-flammability, inexpensiveness and nontoxicity. The main advantage of supercritical fluid technology lies in the possibility of achieving a perfect separation of the supercritical solvent and the processed products since a simple expansion step allows the gas to be recovered and recycled without any further purification [4]. ScCO_2_ possesses other properties such as a low gas-like viscosity, which allows its penetration in many solid substrates and its liquid-like solvent power, which allows many organic compounds to be solubilized. In addition, its solvent power can be easily tuned through density modulation by simply varying either the pressure or the temperature [7].

Given the versatility of scCO_2_ described above, it is not surprising that it has also found many applications in the pharmaceutical area such as particle generation, liposome production, coating, foaming, tissue engineering, aerogel synthesis, drug impregnation, etc. [8,9,10]. Drug complexation with cyclodextrins through supercritical fluid technology is one of these innovative processes that may meet the interests of the pharmaceutical field. In fact, conventional complexation techniques, such as kneading, spray drying, freeze drying, and coprecipitation from various solvents, may suffer some limitations, such as long process time, low encapsulation efficiency, the need of additional drying steps, and high residual organic solvent content [11]. ScCO_2_ is a good candidate as an unconventional complexation medium thanks to its previously mentioned properties. In fact, its residual content in the final products after depressurization is negligible and its mild critical point renders it suitable for complexing thermally labile drugs [8].

The first experiments aiming at encapsulating guest compounds into cyclodextrins using scCO_2_ were conducted in 1990 [12]. Geraniol and mustard oil, which had been previously incorporated into a solid matrix, were extracted by means of a high-pressure (10 MPa) CO_2_ stream, while complexation was achieved after depressurization (5 MPa) by passing the gaseous stream through a solid bed of moistened cyclodextrins. Since then, many other research groups have attempted the encapsulation of different active compounds in the cavity of a cyclodextrin, by employing various techniques, which were first reviewed by Mammucari and Foster in 2008 [13]. However, no other review work can be found in the recent literature even though researchers have kept testing the supercritical fluid complexation (SFC) of many drug/cyclodextrin systems.

This work aims at providing an updated report about the use of scCO_2_ as an unconventional medium to achieve the complexation of many kinds of drugs with native and substituted cyclodextrins. Specific attention is devoted to the description of the different complexation techniques as well as the possible hypotheses about the complexation mechanisms in the supercritical environment and the role of the process working conditions. Differences may arise depending on the fact that the various cyclodextrins may be soluble or insoluble in scCO_2_. Furthermore, the solvent, antisolvent or co-solute role of the supercritical medium in the complexation process should also be considered. The last section of this paper, instead, provides an overview of the different classes of drugs investigated until now and of the main obtained results in terms of complexation efficiency, drug release rate and bioavailability. When possible, the results obtained for the same drug with various cyclodextrins, or different complexation techniques are compared with those obtained with conventional approaches.

## 2. Cyclodextrins Employed in SFC Studies

Different cyclodextrins have been investigated in SFC studies, which can be classified as scCO_2_-soluble and sc-CO_2_-insoluble [14,15,16,17,18,19,20,21,22,23,24,25,26,27,28,29,30,31,32,33,34,35,36,37,38,39,40,41,42,43,44,45,46,47,48,49,50,51,52,53,54,55,56,57,58,59,60,61,62,63,64,65,66,67,68,69,70,71,72,73,74,75,76,77,78,79,80,81,82,83,84,85,86,87,88,89,90,91,92,93,94,95,96,97,98]. These are reported in Table 1, which also reports the acronyms used in this work for their identification. The list includes the three naturally occurring cyclodextrins, αCD, βCD, γCD and some chemically modified derivatives. Naturally occurring cyclodextrins are hydrophilic compounds, which, for this reason are insoluble in the non-polar scCO_2_. The different kinds of moiety employed in the chemically modified cyclodextrins may result in the improvement of their aqueous solubility [99] or, conversely, in providing them good affinity for the supercritical solvent [46,91].

## 3. A Brief Overview of the Techniques Employed to Characterize the scCO_2_-Obtained Formulations

Drug/cyclodextrin formulations obtained through supercritical fluid techniques are characterized by the same tools employed when they are prepared with conventional ones [3,100,101]. Commonly used analytical tools include differential scanning calorimetry (DSC), X-ray diffraction, scanning electron microscopy (SEM) and Fourier-transform infra-red (FTIR) spectroscopy [101]. Common practice is that the spectra or patterns of the pure compounds are compared with those of their physical mixtures and the presumed complex formulations to ascertain if any changes have occurred. X-ray diffraction and SEM are used to identify crystalline structures and investigate morphological changes of the processed materials, respectively, while they do not provide assessment about the true complex formation [101]. Instead, the interpretation of FTIR and DSC analyses is, in most cases, used to ascertain the formation of a complex. While FTIR allows those vibrational modes of the guest and host compounds that are affected during the inclusion process to be identified, DSC provides information about the physical and energetic properties of the samples, which may be a clue of the interactions of these compounds [101]. Even though DSC scan are often used to quantify the inclusion yield of scCO_2_-obtained formulations [29,32,34,43], they do not allow to distinguish if the drug molecules have really been included into the cyclodextrin cavity or have simply been dispersed in the carrier in their amorphous state [101]. For this reason, many authors agree that the best evidence of drug inclusion should be provided by other techniques, such as the “differential solubility method” or nuclear magnetic resonance (NMR) spectroscopy [22,52,63].

The “differential solubility method” [16] allows the total and free drug contents to be determined. It is based on the differential solubility of the drug (soluble) and the cyclodextrin (insoluble) in specific organic solvents (i.e., acetonitrile, cyclohexane, chlorobutane, hexane). When the formulation is contacted with the organic solvent, the included drug remains trapped in the undissolved cyclodextrin, while the non-included drug is dissolved, and the free drug content can thus be assayed by ultraviolet spectroscopy. When the formulation is contacted with a mixture of the organic solvent and water, both the included and free drug are dissolved, and the total drug content can be determined. This technique has widely been employed to assess the inclusion yield of many scCO_2_-obtained formulations [19,22,23,24,25,27,28,38,41,48,53,59,61,62,63,85].

A drawback of the “differential solubility method” is that it does not allow to distinguish between included and non-included complexes. In fact, drug molecules may either enter the cyclodextrin cavity or interact with the external moieties of the glucopyranose units. This point can be overcome by NMR spectroscopy, which can either be conducted in the liquid or in the solid state and can provide direct information about the structure of the complex and of the orientation of the guest molecule inside the cavity of the cyclodextrin [100]. Many researchers adopted this tool to support the inclusion complexation of different drugs in cyclodextrins obtained through supercritical fluid techniques [20,40,41,45,46,49,55,56,79,83,85,91,92,94,95,96,97]. Furthermore, in some cases, the possible host/guest interactions during complexation were deepened by means of molecular modelling such as docking studies [23,57,79,87,89] and molecular dynamics [57,95,102]. The possibility of distinguishing between inclusion and non-inclusion complexes was debated in the literature especially as far as the processing of scCO_2_-soluble cyclodextrins is concerned (Section 4.4).

The efficacy of the obtained drug/cyclodextrin formulations can be tested with in vitro and in vivo tests commonly employed in pharmaceutics [3]. In vitro drug release profiles of the obtained formulations are generally conducted in stirred aqueous solutions at different pH values and compared with those of the pure drug and the drug/cyclodextrin physical mixtures. This approach is widely used in the great majority of research papers investigated in this work [11,19,21,22,25,27,28,29,30,31,34,39,41,42,43,44,46,48,49,50,51,52,53,54,55,56,57,58,60,64,65,67,68,69,70,71,72,75,76,79,80,81,84,86,87,88,89,91,92,93,94,96,98]. On the other hand, unfortunately not many works report in vivo tests whose details can be found in the specific references [25,49,65,69,71,72,75,80,81]. Other works report different analyses to detect the biological performance of the formulations, such as the evaluation of the antifungal, antibacterial or antioxidant activity [40,67,77,80] as well as in vitro cell culture assays [73] or taste perception tests [46]. Two works report specific studies to test the stability over time of the complexes prepared through supercritical fluid techniques [22,45].

As a conclusion of this section, it must be pointed out that many works compared the scCO_2_-obtained formulations with those prepared with conventional techniques [18,27,30,31,33,36,40,41,45,46,49,53,54,57,59,61,62,64,67,68,85,88,89,98], such as coprecipitation, heat-sealing, co-grinding, freeze drying, kneading, etc. The comparison was performed either in terms of sample characterization or in terms of drug release profiles or both, depending on the specific research report.

## 4. Complexation Through Supercritical Solvent Impregnation

**Figure 2:**
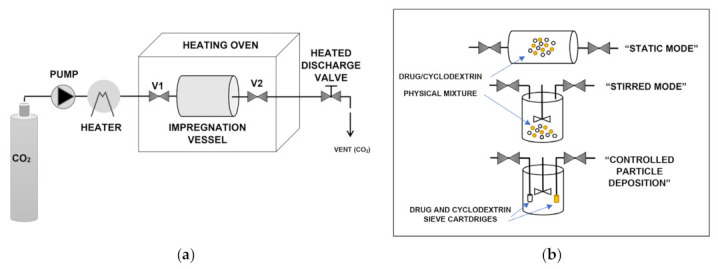
The SSI technique: (**a**) Simplified scheme of the general batch process; (**b**) Details of the three different configuration modes for the impregnation vessel that are available in the literature.

Supercritical solvent impregnation (SSI) techniques are based on the solubilization ability of scCO_2_ either versus the drug or the cyclodextrin and aim at achieving host-guest complexation at supercritical conditions before depressurization. Figure 2a depicts a simplified scheme of the general SSI batch process. The drug and the cyclodextrin are placed into an impregnation vessel and contacted with scCO_2_ at constant temperature and pressure for a fixed period (valves V1 and V2 closed). The procedure is terminated by rapid decompression to atmospheric pressure, which involves CO_2_ gasification and its separation form the obtained complex. The different setups available in the literature differ for the type and operation mode of the impregnation vessel (Figure 2b). The simplest and most used technique is the “static mode”, which consists in placing a physical mixture of the drug and the cyclodextrin at pre-defined molar ratios in the impregnation vessel and in contacting it with scCO_2_ without any agitation [16,20,21,22,24,37,40,44,45,58,63,64,65,66,67,85,88,95]. This procedure was also named by some authors as supercritical “maturing” or “maturation” process [32,52].

A simple variation of the “static” procedure is the “stirred mode”, which consists in contacting the physical mixture, set inside the impregnation vessel, with scCO_2_ under mechanical or magnetic stirring [17,26,39,53,91,94,96,98]. The “stirred mode” is preferred to the static one especially when scCO_2_-soluble cyclodextrins are involved [26,91,94,96,97,98].

Other authors proposed another variation of the SSI batch technique [27,28,29]. They placed the drug and the cyclodextrin into separate cartridges with a sieve form, which were then positioned inside a stirred high-pressure cell under a supercritical atmosphere. This procedure was named “controlled particle deposition” and was employed by the authors both with powder βCD [27,28] and βCD granules for oral delivery [29]. Similarly, another research group [60] impregnated a HPβCD-functionalized polymeric membrane with ibuprofen by keeping the membrane and the drug into two different compartments during the SSI procedure.

As it has been previously mentioned, the SSI process can be conducted either with scCO_2_-insoluble and scCO_2_-soluble cyclodextrins. The solubility or insolubility of the cyclodextrin in the supercritical solvent determines different complexation mechanisms, which are discussed in Section 4.1. As far as scCO_2_-insoluble cyclodextrins are concerned, to increase the complexation efficiency, different working conditions, such as temperature and pressure of the SSI process have widely been explored. Furthermore, auxiliary agents such as acids, amino acids, polymers, water, or their combinations can often be added [19,22,32,34,38,43,52,63]. Another strategy is to employ supercritical mixtures of CO_2_ and a cosolvent such as ethanol or water [17,18,37,39,44]. In Section 4.2 and Section 4.3 the role of the working conditions (temperature and pressure) as well as that of auxiliary agents and cosolvents on the complexation efficiency of scCO_2_-insoluble cyclodextrins is discussed.

### 4.1. ScCO_2_-Insoluble Cyclodextrins: Hypotheses about the Complexation Mechanism

ScCO_2_-insoluble cyclodextrins are the most employed ones in the literature and a possible drug-inclusion mechanism at supercritical conditions was proposed ever since the first experiments were conducted. According to Van Hees and co-workers [16] it is commonly accepted that the general mechanism of inclusion of a guest molecule into the internal cavity of a cyclodextrin is mainly a substitution of the naturally included water molecules by a less polar guest. This could be probably promoted by scCO_2_, especially at high temperature and pressure since the higher solubility of water in the supercritical medium would help them escape from the cyclodextrin cavity [16]. Evidence of this theory was supported by the fact the DSC curves of pure cyclodextrins treated with scCO_2_ revealed a reduction of the water content with respect to the untreated compounds [21].

The facilitated escaping of water from the cavities of the cyclodextrins, however, is not enough to explain complexation since drug/cyclodextrin physical mixtures kept in oven at the same temperature exhibited dehydration but not complexation [16]. It was, then, suggested that a reaction with different steps would also occur between the cyclodextrin and the drug. While the cyclodextrin is insoluble in the supercritical solvent, the drug needs to be soluble before its inclusion but, once included, it would no longer be soluble, and the equilibrium would continually be displaced towards the complex [16]. According to other authors [27,53], the scCO_2_-dissolved drug would be simply transported inside the carrier and be there precipitated upon depressurization so achieving its inclusion in the cyclodextrin cavity. Under these hypotheses both the solvating power and the high diffusivity of scCO_2_ would play a key role in the inclusion mechanism and their combination could help maximize the complexation efficiency [53]. Anyway, independently from the hypothesized mechanism, inclusion and/or molecular dispersion of the drug in the carrier is possible thanks to specific molecular interactions, such as hydrogen bonding or hydrophobic interactions, between the drug and the cyclodextrin [58].

In addition to the above-described possible mechanisms, the well-known phenomenon of the melting point depression caused by scCO_2_ towards some organic compounds is considered by some authors to enhance the complexation efficiency [59,61,62,84,86,87,88,89]. The melting point of a solute decreases due to the CO_2_ sorption into its solid matrix and the consequent solvent-solute interactions, which result in weaker interactions between the solute segments within the matrix [84].

Melting point depression of drug molecules, such as piroxicam [16,37], ibuprofen [28] and naproxen [103] is reported in the literature. Furthermore, the melting point depression of MβCD and its consequent liquefaction was also observed [59,84]. The melting point of MβCD decreased from 110 °C to 70 °C when contacted with scCO_2_ at 8 MPa [59], while it decreased to 25 °C if the pressure was raised up to 19 MPa [84]. On the other hand, the liquefaction phenomenon was not observed for other cyclodextrins when exposed to the supercritical solvent [59].

The molten state of one or both components upon scCO_2_ treatment was considered a possible explanation for a successful drug-cyclodextrin inclusion complexation [36,58]. He [59], for example, compared the SFC of shikonin with MβCD and HPβCD. Complete complexation of shikonin and MβCD was found while quite low efficiency was observed with HPβCD. According to the author, complexation would occur due to two mechanisms: the drug dissolution in scCO_2_, with the consequent impregnation of the cyclodextrin carrier, and the direct dissolution of the solid drug in the cyclodextrin melt. Since HPβCD did not exhibit any melting point depression, its complexation with shikotin was quite low since only one of the above-cited mechanisms would occur. On the other hand, the melting point depression of MβCD allowed the shikotin/MβCD to achieve complete complexation [59] thanks to the occurrence of both mechanisms, where the second was the most efficient. Similarly, complete inclusion results were obtained with MβCD by other authors [61,62,86,87,88,89].

### 4.2. ScCO_2_-Insoluble Cyclodextrins: Role of Temperature and Pressure on Complexation Efficiency

The temperature and pressure ranges of the SSI process may vary between 35 to 160 °C [19,37,84] and 10 to 45 MPa [16,24,89], respectively, and the choice of the most appropriate conditions strongly depends on each drug/cyclodextrin system. Their role on the complexation efficiency and/or drug amorphization is quite complex since contrasting reports can be found in the literature. Most research works agree with the conclusion that a temperature increase is beneficial to drug/cyclodextrin complexation [16,19,21,24,30,32,33,34,38,52,63] while only a couple of papers [44,53] reported the opposite experimental trend. Even though the thermodynamics of complexation in scCO_2_ had not been specifically investigated, some researchers [34] related the positive effect of temperature to a favorable shift in the equilibrium of complexation towards the complexed form. Other authors, instead, explained it with an acceleration of the kinetics of complexation [32].

As far as pressure is concerned, while some research reported that its role on the complexation efficiency can be considered negligible [16,63], others reported that its increase may have a positive [21,84] or a negative effect [19], respectively. Furthermore, some authors reported that the pressure increase was beneficial only above a certain temperature level [24] or that the complexation yield first increased versus pressure until a maximum was reached and then decreased [44,61,66]. Some authors, instead, preferred to discuss how the scCO_2_-density, rather than pressure, affected the complexation yield [32,41,52]. However, also in this case, contrasting results were obtained and the solvent density was found to increase [32] or to decrease [41,52] the complexation efficiency.

Temperature and pressure strongly affect the drug solubility in the supercritical medium and their variation may also determine the liquefaction either of the drug or the cyclodextrin. This last phenomenon may enhance the complexation efficiency as it was explained in Section 4.1. As far as the solubility of the drug in scCO_2_ is concerned two aspects should be considered. The first one is that, even though the drug solubility in scCO_2_ is essential to achieve the SSI of the carrier [16], a too high solubility level may decrease the inclusion yield since the drug would leave the cyclodextrin cavity to dissolve in the CO_2_ phase [41,62], which would exert a diluting effect [32]. The second aspect is related to how temperature and pressure affect the solubility of a solute in scCO_2_, which is a phenomenon well-known in the literature [53,104]. At constant temperature, in fact, the solubility of a solute always increases with pressure since the density and solvent power of the fluid increase. At constant pressure, instead, a temperature increase exhibits two competitive effects: on one hand it increases the vapor pressure of the solute so resulting in solubility enhancement, on the other it decreases the density and solvent power of the fluid, which involves a reduction in solubility. The first effect prevails on the second above a solute-specific pressure value, which is known as the “cross-over point”, while the opposite occurs below.

A tentative explanation of the so different experimental trends observed for the complexation efficiency versus temperature, pressure and solvent density should consider that they are the result of those different concomitant phenomena that have been previously discussed. The choice of the pressure range with respect to the “cross-over point” determines if a temperature raise is beneficial to the solubility of the drug in the solvent medium or not. On the other hand, the effective extent of the drug solubility in scCO_2_ and the solvent amount in the impregnation vessel determine if the CO_2_ can be an appropriate vehicle for cyclodextrin impregnation or can, instead, exert a diluting effect. In the first case, for example, a pressure increase would enhance the complexation efficiency while in the second the opposite result would be obtained since the drug would be too soluble in the solvent phase. The whole mechanism is complicated by the fact that a temperature change could affect the kinetics or shift the equilibrium of complexation [32,34]. Furthermore, the occurrence of liquefaction phenomena for one or both components could give rise to a second complexation route in addition to supercritical impregnation.

### 4.3. ScCO_2_-Insoluble Cyclodextrins: Role of the Auxiliary Agents and Cosolvents on Complexation Efficiency

The addition of auxiliary agents is a common practice in conventional complexation techniques since it increases the drug inclusion efficiency into cyclodextrins. This is advantageous because it allows the desired solubilizing or stabilizing properties of the drug to be achieved by employing lower amounts of cyclodextrins, whose content in pharmaceutical formulations may be limited by their potential toxicity, high costs or dosage problems [99]. Auxiliary agents such as water, polymers, basic and acidic compounds can be employed also with many drug/cyclodextrin systems processed by SSI [19,22,32,34,38,43,52,63]. The procedure is simple and consists in adding one or more auxiliary compounds to the drug/cyclodextrin physical mixture before contacting it with the supercritical solvent.

The addition of water as an auxiliary agent was investigated to increase the complexation efficiency of some drug/cyclodextrin systems [22,32,34,38,43,52,63]. As has already been mentioned [16], cyclodextrins are naturally hydrated and their cavities contain water molecules, which are substituted with less polar guest molecules during complexation. Water addition to physical mixtures generally consists in increasing the natural moisture content, which ranges from 6 to 15% (*w*/*w*) [32,34,38,63], up to 25–27.5% (*w*/*w*), which is the maximum limit before obtaining a paste [34,38,63]. Water addition was always beneficial and in many cases the complexation efficiency increased from low or negligible levels up to complete or almost complete inclusion [32,43,52].

Fages and coworkers [32,52] proposed a mechanism to explain the benefit of water addition as an auxiliary agent in the SSI process. They hypothesized that, due to water-saturation of scCO_2_, part of the water added to the physical mixture could remain in its liquid state on the surface of the solid partially dissolving the cyclodextrin. The partial solubilization of the cyclodextrin would promote surface cracking and consequently increase the surface of contact. This would destabilize the water initially contained in the cyclodextrin cavities, which could be more easily replaced by the drug molecules [32]. The theory was supported by measuring, through DSC thermograms, the heat of dehydration of βCD before and after SSI. It was found that the water remaining in the complex after the supercritical treatment was adsorbed on βCD with weaker bonds with respect to that present in the initial physical mixture [32].

The addition of water-soluble polymers as auxiliary agents is a common strategy in conventional complexation techniques [99]. Their addition can either increase the complexation efficiency of the drug into the cyclodextrin cavity or promote the amorphization of crystalline drugs, which results in higher solubility in aqueous media [22,99]. Similar results were also obtained by adding polyvinyl pyrrolidone (PVP) in the SFC of piroxicam with HPβCD [63].

Another common conventional method to increase the complexation efficiency is the addition of an acidic ternary compound when a basic drug is employed, or, conversely, a basic ternary compound when an acidic drug is used [22,99]. As far as basic auxiliary agents are concerned, L-lysine or trometamol were successfully employed by different authors [16,34,38,63] in some cases also in combination with water [34,38,63]. Specific studies to investigate the role of these basic auxiliary agents on the complexation efficiency in a supercritical environment were not performed. As far as L-lysine is concerned it was either hypothesized that it could enhance the mechanism of drug replacement of the water molecules initially included in the cyclodextrin cavities [34] or allow a ternary complex to be formed thanks to the formation of a salt between the drug and the aminoacid [38]. On the other hand, different acids (citric, fumaric, tartaric, malic, maleic acid) were employed to investigate their role on the SFC of miconazole with different cyclodextrins [22]. Successful improvement of the complexation yield was obtained, which strongly depended on the most appropriate combination of the drug with the acid and cyclodextrin. Subsequent studies [23,35,82] were carried out to better understand how supercritical inclusion was formed and a possible mechanism was proposed. It was proposed that, on one hand a drug/acid/cyclodextrin ternary complex may be formed; on the other, an acid/cyclodextrin binary complex may also be formed, which hinders the inclusion of the ternary one. Depending on the specific drug/acid/cyclodextrin combination, one mechanism may prevail over the other so determining the successful or the unsuccessful inclusion of the drug [35].

Adding a cosolvent to scCO_2_ is a different strategy to increase the yield of complexation. This is common practice in supercritical extraction or impregnation processes to enhance the solubility of solutes in the supercritical environment. In this context, water could be employed as a cosolvent [39,42,44] instead of being added to a drug/cyclodextrin physical mixure as an auxiliary agent. However, from the exam of some literature works involving the ketoprofen/βCD system [39,42] it can be concluded that using water as a cosolvent leads to results similar to those obtained when water is added to the physical mixtures as an auxiliary agent [32]. Other experiments reported the use of different cosolvents, and the use of ethanol was particularly successful [17,18,37]. Grandelli and coworkers [37], who investigated the piroxicam/βCD system, explained that the addition of a cosolvent may favor the melting point depression of the drug. This may increase the complexation efficiency as well as avoid the degradation of thermally labile drugs, such as piroxicam.

### 4.4. ScCO_2_-Soluble Cyclodextrins: Hypotheses about the Complexation Mechanism

Hydrophobic cyclodextrins are employed in the literature as sustained release carriers especially for water-soluble drugs [92]. Their hydrophobic nature confers them good solubility in scCO_2_, which allows them to be easily used to encapsulate drugs by SSI.

If both the drug and the cyclodextrin are soluble in scCO_2_, SSI is easily achieved. Two possible complexation mechanisms were proposed. According to Tozuka and coworkers [26,90], complexation occurs because the solubility of the complex in scCO_2_ is lower than that of the uncomplexed components, which would result in the precipitation of the complex from the supercritical medium [90]. Recently, another research group [96] proposed a different complexation mechanism, which is based on the experimental measures of the cloud point of both components. They supposed that the drug and the cyclodextrin would first dissolve and complex in the supercritical medium. Then, as soon as the pressure is released, the cyclodextrin, which exhibits higher cloud point pressure, would first precipitate along with the complexed drug while the uncomplexed drug would remain in solution and be vented off the system during the subsequent decompression [96].

Successful complexation experiments of scCO_2_-soluble cyclodextrins were also performed with hydrophilic drugs, which exhibit poor or negligible solubility in scCO_2_ [91,92,93,97]. No research group proposed a possible complexation mechanism in this case, however Lee and coworkers [92] reported interesting visual observations during the SSI of molsidomine and PAβCD. Cloud point experiments were conducted in a vessel equipped with a sapphire quartz window that allowed visual observations. Even though molsidomine alone was found to be insoluble in scCO_2_, after the addition of PAβCD in the vessel, the solid drug slowly disappeared and the whole system achieved homogeneity after 30 min under stirring at 34.5 MPa and 45 °C [92]. This demonstrates that complexation occurred and resulted in the solubilization of an insoluble drug in scCO_2_.

Provided that scCO_2_-soluble cyclodextrins can complex both with scCO_2_-soluble and sc-CO_2_-insoluble drugs by means of SSI, researchers have debated if host-guest inclusion complexes are formed or not. According to Ivanova and coworkers [97], who investigated the complexation of TAβCD with two different drugs, non-inclusion complexes were formed with the drug probably interacting with the external acetyl groups of the cyclodextrin instead of entering its cavity. This was proved by high-pressure NMR studies of the TAβCD dissolved in scCO_2_. It was found that the acetyl groups, which allow the TAβCD to be solubilized in scCO_2_, give rise to structural changes of the dissolved cyclodextrin with a consequent self-closure of the glucopyranose cavity that affects its inclusion capability in the supercritical medium. This self-closure of the molecular cavity and the consequent reduction of its accessibility to guest molecules was observed for other drug/cyclodextrin systems through molecular dynamic simulations [102] and NMR studies [96]. On the other hand, NMR investigations of omeprazole/PAβCD complexes obtained by SSI supported the hypothesis of host-guest inclusion interactions [94].

A possible answer to the inclusion/non-inclusion debate can be found in the work by Ingrosso and coworkers [95] who compared the complexation of benzoic acid, toluene, and benzoic aldehyde with PAβCD. This study combined solid-state NMR investigations with molecular dynamics simulations. While toluene and benzoic aldehyde left the cyclodextrin cavity, benzoic acid remained within the cavity all along the simulation time and a stable inclusion complex of this last compound with PAβCD was experimentally obtained. The authors hypothesized that the formation of hydrogen bonds is the driving force leading to host-guest complexation even though the self-closure of the cyclodextrin cavity reduces its accessibility in the supercritical medium. The presence of polar guest molecules with hydrogen-bond donor capacity would, then, succeed in forcing the opening of the cyclodextrin cavity so achieving stable inclusion complexation [95].

## 5. Complexation Through Particle-Formation Techniques

The supercritical fluid technology can be used to achieve particle formation and many examples of novel pharmaceutical manufacturing processes can be found in the literature [8]. The processes can be categorized based on the different role of scCO_2_, which can act as a solvent, an antisolvent or a co-solute [8,105]. Even though many scCO_2_ soluble cyclodextrins are reported in the literature, until now examples of particle-formation techniques where scCO_2_ acts as a solvent have not been employed to achieve drug/cyclodextrin complexation. On the other hand, examples where scCO_2_ is employed either as an antisolvent [11,46,47,48,49,50,51,54,55,56,68,69,70,71,72,73,74,75,76,78,79,80] or a co-solute [57,77,83] can be found. These techniques match the possibility of achieving drug/cyclodextrin complexation with that of controlling the particle size distribution [49,54,68,73,77,79,83] which may also positively affect the dissolution rate of the obtained products. Furthermore, even though organic solvents need to be employed, these processes allow easy single-step removal of their residuals [68], whose final content in formulations were proved to be much lower than those limits suggested by different national and international authorities [46,50,72,79].

### 5.1. Particle-Formation by Using scCO_2_ as an Antisolvent

The supercritical antisolvent (SAS) processes exploit the poor solubility of active compounds in scCO_2_. Organic solvents, such as ethanol, methanol, acetone, dichloromethane (DCM), dimethyl sulfoxide (DMSO), dimethylformamide (DMF) are used to dissolve the drug and the cyclodextrin, which are then mixed with scCO_2_. During the process, the mixture expands to supersaturation and scCO_2_ acts as an antisolvent so causing the precipitation of the complexes from the organic solvents [6,8]. To be successful a SAS process requires that the scCO_2_ and the selected solvent are completely miscible, while the drug and the carrier have to be soluble in the solvent but insoluble in the scCO_2_-solvent binary mixture [50].

Drug/cyclodextrin systems were processed with the gas antisolvent (GAS) process [56,75,78], the aerosol solvent extraction system (ASES) [11,46,50,68,71,72,76,80], the solution-enhanced dispersion by supercritical fluids (SEDS) [47,48,49,51,54,55,69,70,79] and the atomized rapid injection solvent extraction (ARISE) system [73,74], even though the general acronym SAS is often used to indicate any of the above-cited variations.

**Figure 3:**
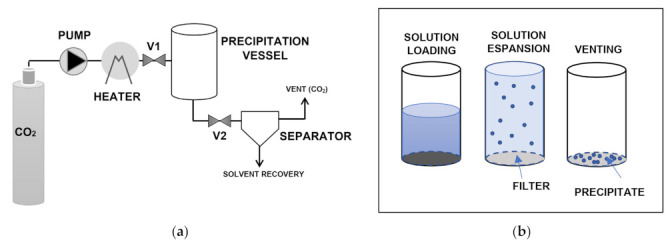
The GAS technique: (**a**) Simplified scheme of the process; (**b**) Schematic of the different process steps in the precipitation vessel.

GAS is a batch process whose simplified scheme is reported in Figure 3. First, the liquid solution containing the drug and carrier is loaded in the precipitation vessel. Liquid CO_2_ is then pumped and heated up to the selected supercritical conditions through valve V1 (valve V2 is closed). During pressurization, the liquid in the precipitation vessel is expanded, supersaturation is achieved, and the solutes are precipitated. After, valve V2 is opened, the vessel is depressurized, and a separator allows the solvent to be recovered from the gaseous CO_2_. Finally, the obtained particles are collected from the filter located at the bottom of the vessel [6].

**Figure 4:**
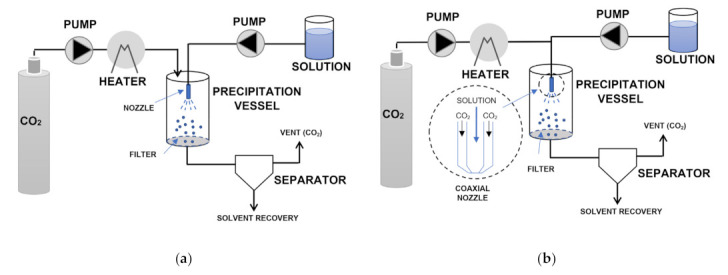
(**a**) Simplified scheme of the ASES/PCA process; (**b**) Simplified scheme of the SEDS process.

ASES, which is also known as the precipitation with compressed antisolvent (PCA) method, is a semicontinuous process (Figure 4a). This process generally leads to precipitated particles with smaller size and stricter particle size distribution with respect to GAS [105]. ScCO_2_ is continuously introduced at a fixed flowrate into the precipitation vessel, which is maintained at constant temperature and pressure. Then, the solution is injected into the pressurized vessel at constant flowrate through an atomization nozzle so that supersaturation and the consequent precipitation of solutes are achieved.

After the solution has been completely delivered, the scCO_2_ is kept flowing to remove residual solvent while a separator provides CO_2_/solvent separation. Eventually the particles are collected at the bottom of the vessel after pressure relief [6]. SEDS is a modified version of ASES/PCA (Figure 4b). The main difference lies in the atomization device, which consists in a coaxial nozzle that provides the simultaneous introduction of the solution and the supercritical solvent [6]. This involves a tangential impact between the two fluids, which promotes better mixing so leading to the production of finer powders and preventing particle aggregation [6,105].

**Figure 5:**
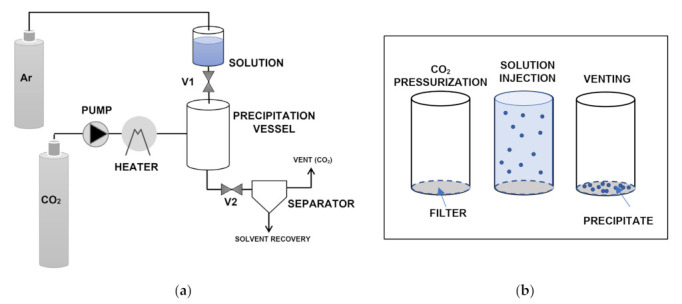
The ARISE technique: (**a**) Simplified scheme of the process; (**b**) Schematic of the different process steps in the precipitation vessel.

The ARISE method was proposed to circumvent the problems connected with the use of nozzles [73,74]. Nozzle size and type, in fact, strongly affect mixing hydrodynamics as well as nucleation and growth rate of the precipitates so influencing the characteristics of the final products. Unfortunately, the above cited conditions are difficult to be reproduced in the scale-up of the process, which may limit the application of SAS technologies at larger scale [73]. Furthermore, micrometric nozzles may give rise to blockage during operation, which is a very common problem coming out from the use of nozzles in supercritical assisted processes. [73]. ARISE is a batch process that exploits a pressure difference to achieve effective mixing between the solution and the antisolvent (Figure 5). The precipitation vessel is first pressurized with scCO_2_ (valves V1 and V2 closed) while the solution is charged into an upper vessel that is pressurized with argon. After a pressure difference is established between the two vessels, valve V1 is opened and rapid injection of the solution in the precipitation vessel is achieved, which leads to solutes precipitation. Eventually (valve V2 open), scCO_2_ cleaning and solvent recovery can be performed similarly to other SAS methods [73,74].

The use of SAS techniques with drug/cyclodextrin systems generally produces micrometric or nanometric complex particles with narrow size distributions and spherical morphology [11,46,47,48,49,50,51,52,55,68,71,72,76,79,80], which display faster and more reproducible drug dissolution rates with respect to those of the unprocessed drugs or their physical mixtures during in vitro release tests. The increase of the dissolution properties can be ascribed both to the formation of inclusion complexes and to the reduction of the particle size [49]. As far as the size and morphology of the precipitated particles are concerned, these are a complex result of different operating parameters such as scCO_2_ temperature and pressure, solvent concentration, nozzle diameter etc. [6]. In particular, the higher the pressure in the precipitation chamber the smaller is the particle size, which may range from the micro- to the nanoscale [105]. Reverchon and coworkers [106] have recently reported a comprehensive discussion of the coprecipitation mechanism in SAS processes and the role of the working conditions on particle size and morphology, which can be applied also to drug/cyclodextrin formulations [48,51].

The mechanism of drug/cyclodextrin complex formation in SAS processes have not been deeply investigated in the literature and they are still open to discussion. Complexes may be formed either in the liquid solution before contacting the supercritical antisolvent, or during the subsequent precipitation [51]. If the second hypothesis would occur, complexation could be favored at higher pressure and lower temperature (i.e., higher scCO_2_ density) [80]. In fact, since higher scCO_2_ density increases the solution supersaturation, this would result in the formation of smaller drug particles, which could more easily enter the cyclodextrin cavity during precipitation [80]. Other authors [48], instead, observed an experimental increase of the inclusion yields versus temperature and related this phenomenon to the favorable shift in the equilibrium of complexation towards the complexed form. Successful or unsuccessful complexation may also be related to the major or minor solubility of the drug in scCO_2_. If this is too high the dissolved drug would escape before complexation; if it is too low, instead, the drug would precipitate before the complexation takes place. This was supported by observing that the inclusion yield of budesonide in γCD was inversely correlated with the solvent power of scCO_2_ [54].

The above discussion has pointed out how the mechanism of drug complexation during SAS precipitation is far from being elucidated. The crucial point is that drug complexation (if occurs) is probably concomitant to particle precipitation, and this makes it difficult or even impossible to investigate the two phenomena independently. In addition, both phenomena have the same (beneficial) effect on the obtained formulations, which consists in improving the drug aqueous solubility and dissolution rate. Another aspect is that, to the author’s knowledge, no investigation has still been performed to point out if the differences in the operation mode of the various SAS methods (GAS, ASES, SEDS, ARISE) may significantly affect the complexation mechanism.

### 5.2. Particle-Formation by Using scCO_2_ as a Co-Solute

Different supercritical fluid technologies employ scCO_2_ as a co-solute to achieve particle formation for pharmaceutical purposes [105]. Among these, supercritical-assisted atomization (SAA) has recently been employed to prepare drug/cyclodextrin complexes [57,77,83]. The SAA method, which is also known as supercritical-assisted spray drying (SASD), is a valid alternative to the conventional spray drying process.

**Figure 6:**
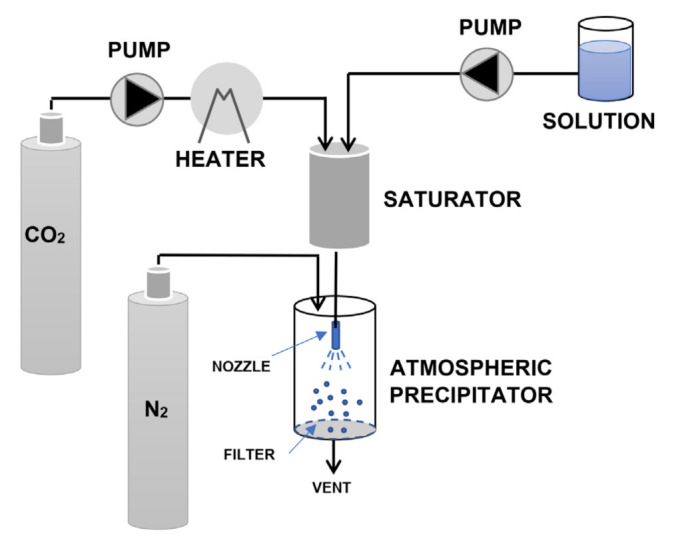
Simplified scheme of the SAA/SASD process.

In SAA, the dissolved scCO_2_ is employed as a pneumatic agent to generate fine particles through atomization [8]. It is based on the dissolution of a controlled amount of scCO_2_ in a solution containing the components to be precipitated, which is followed by spray drying at atmospheric conditions [105]. A simplified scheme of the SAA/SASD process is reported in Figure 6. An alcoholic or hydroalcoholic solution containing the drug and the cyclodextrin is contacted with scCO_2_ in a saturator (packed column) to achieve mixing and complete solubilization of the CO_2_. Then, the expanded liquid is injected by means of a nozzle into an atmospheric precipitation vessel in the presence of hot nitrogen, which provides complete evaporation of the employed liquid solvents [105]. Two phenomena occur to achieve particle formation: the generation of primary droplets at the exit of the nozzle and the fast release of the dissolved CO_2_ upon decompression. The two phenomena are known as “pneumatic atomization” and “decompressive atomization”, respectively [105]. More details about the possible micronization mechanism and the role of the different operating conditions in process design can be found in the specific literature [9,105].

Even though examples of pure cyclodextrin micronization by SAA date back to 2006 [107], few experiments in the presence of drugs have been reported and only in very recent years [57,77,83]. Due to the limited amount of research, no specific studies or hypotheses can be found about the possible complexation mechanism occurring during SAA precipitation. It was only suggested that the scCO_2_ dissolved in the liquid solution could promote specific intermolecular interactions between the drug and the carrier, which could result in the modification of their physicochemical and physiologic properties [83].

## 6. Complexation Results Obtained with Different Classes of Drugs

A huge number of drugs have been processed with different kinds of cyclodextrins through supercritical fluid techniques over the last years. This section aims at providing an overview of the main obtained results. The investigated drugs are discussed in four different sections. The first and the second section include non-steroidal anti-inflammatory drugs (NSAIDs) and antifungal drugs, respectively. The third section reports the results obtained with essential oils and other natural compounds. The last section groups all the drugs that have not been included in the previous sections.

The various drugs have been complexed with scCO_2_-insoluble and scCO_2_-soluble cyclodextrins, which depend on the drug-delivery purpose. ScCO_2_-insoluble cyclodextrins are generally employed to enhance the drug release rate and bioavailability of poorly-water soluble drugs while scCO_2_-soluble cyclodextrins can be employed to slow down the dissolution rate of drugs aiming at sustained release performance of the formulations.

### 6.1. Non-Steroidal Anti-Inflammatory Drugs (NSAIDs)

Most NSAIDs are good examples of poorly-water soluble drugs, which may require both a fast and a prolonged release depending on the type of inflammation to be treated. Thanks to their solubility in scCO_2_, many NSAIDs are widely processed with scCO_2_ for pharmaceutical purposes [108]. Table 2 summarizes the main research activities conducted on this class of drugs as far as SFC is concerned. Among the different drugs, ibuprofen, ketoprofen and piroxicam were explored by different research groups and often employed to investigate the complexation mechanism at supercritical conditions. Flurbiprofen, instead, is the only NSAID for which in vivo release studies can also be found [65].

#### 6.1.1. Flurbiprofen

Literature reports of flurbiprofen complexation by SSI with TMβCD [90], MβCD [87] and HPβCD [65] can be found. While complexation of this drug with TMβCD was hypothesized thanks to the appearance of a new crystalline phase in the X-ray spectra and DSC scans [90] its complexation with MβCD was, on the contrary, supported by sample amorphization as well as molecular docking studies [87]. The flurbiprofen/MβCD system was also subjected to in vitro dissolution studies, which showed that the dissolution properties of the drug were much improved after the supercritical treatment: while only 1% of the unprocessed drug was released after 60 min, up to 99% of the drug was dissolved from the scCO_2_-obtained complexes after 30 min.

The complexation of flurbiprofen with HPβCD is a complete investigation example, which also includes in vitro and in vivo release studies [65]. Inclusion complexes were prepared by SSI and a conventional co-lyophilization method, which was employed as a reference. The scCO_2_-obtained samples displayed higher complexation efficiency (82%) with respect to those obtained with co-lyophilization (69%). In vitro studies revealed an instantaneous pH-independent release of the drug. Both flurbiprofen and flurbiprofen/HPβCD complexes were introduced in hydrophilic gels to perform in vivo percutaneous permeation studies. The in vivo pharmacokinetic studies showed a relative bioavailability comparable to that of a commercial product as well as a 2-fold increase in C_max_ and a shortened T_max_, which could provide improved pharmaceutical application of this drug.

#### 6.1.2. Ibuprofen

Ibuprofen was complexed with various cyclodextrins and different supercritical fluid techniques. While its complexation with βCD by SSI failed at 35 °C and 12 MPa [26], inclusion yields ranging from 50 to 88% were obtained by operating at higher temperature and pressure (40 °C, 25–30 MPa) [27,28]. The dissolution studies in solutions at different pH resulted in faster drug release of the scCO_2_-obtained complexes with respect to physical mixtures or the unprocessed drug [28]. The release performance was comparable to that of complexes prepared by means of a conventional freeze-drying method [27].

Ibuprofen was complexed with chemically modified cyclodextrins such as MβCD and HPγCD [83,84]. The complexation with MβCD by SSI resulted in formulations with instantaneous release in water solutions: the dissolution rate coefficient was at least 66 times higher than that of the corresponding physical mixture [84]. The successful result was ascribed to complete drug complexation, which was favored by the melting point depression of MβCD (Section 4.1) during SSI. Ibuprofen complexation with HPγCD was instead performed by SAA/SASD [83]. Thanks to the combination of different analytical techniques (DSC, X-ray diffraction, FTIR and NMR spectroscopy), the formation of amorphous inclusion complexes could be assessed even though no drug dissolution studies were performed to investigate the practical efficiency of the obtained formulations.

ScCO_2_-soluble cyclodextrins such as DMβCD, TMβCD and PAβCD [26,90,96] were also processed with ibuprofen by SSI. Ibuprofen complexed with TMβCD after the supercritical treatment since DSC and X-ray diffraction revealed the disappearance of the drug crystals and the appearance of a new crystalline phase, which was assigned to the presence of a complex [26,90]. According to the same authors [26], ibuprofen/DMβCD, instead, only resulted in amorphous formulations and the occurrence of complexation could not be demonstrated. Unfortunately, no release tests were performed neither with the ibuprofen/DMβCD or ibuprofen/TMβCD system to ascertain if the obtained formulations could allow a sustained release of the drug to be obtained. On the other hand, dissolution studies of ibuprofen/PAβCD complexes obtained by SSI were performed [96]. The complexes resulted in a retarded release rate since 33% of the drug was released after 5 min, with respect to the 86–94% amount of drug released by the physical mixture and unprocessed drug after the same time. However, the drug release from the complex was almost complete (94%) after 60 min, which forced it to be still classified as “immediate release”. The authors explained this result pointing out that non-inclusion complexes were formed, which was supported by NMR spectroscopy. The drug/cyclodextrin interactions in non-inclusion complexes are, in fact, weaker than those of the inclusion ones, which would explain the limited retarding effect of drug release [96].

Ibuprofen was not only processed with powder cyclodextrins. The drug was loaded, by means of SSI, into preformed βCD granules ready for oral delivery [29]. The results were compared with those obtained with a conventional solution immersion method where hexane was employed. The SSI-processed samples resulted in a much higher drug loading (17.5 wt.% vs. 4 wt.%). Both techniques resulted in comparable increase of the dissolution rate of the drug in a pH 6 buffer solution with respect to unprocessed ibuprofen, thanks to the almost complete drug inclusion/amorphization in the formulations. Other authors [60], instead, developed a poly methyl methacrylate (PMMA) membrane functionalized with HPβCD for the controlled release of ibuprofen. The membrane was prepared through scCO_2_-assisted phase inversion method while the drug was subsequently loaded by an SSI technique. The entrapment of the cyclodextrin in the polymer did not affect the drug loading but affected its release rate in a pH 7.4 buffer solution. After 40 days the drug release from the functionalized membraned was up to 6 times higher with respect to the neat membrane. This significant result was explained either with a higher hydration action of HPβCD towards the polymer matrix or the facilitated access of the release medium in the drug-containing cyclodextrin cavities [60].

#### 6.1.3. Ketoprofen

A few papers are reported in the literature [32,38,39,42,43] as far as ketoprofen complexation with natural βCD by SSI is concerned. They all agree that adding extra water with respect to natural cyclodextrin humidity is essential for complexation to occur. Water can be added as an auxiliary agent to the physical mixture before the supercritical treatment [32,38,43] or as a cosolvent in the scCO_2_ atmosphere [39,42]. In both cases complexation can be obtained. However, the extent of the complexation yield is a matter of debate. In fact, while two research groups [32,39,42] determined complete or almost complete complexation yields by means of DSC scans, another group [38] employed the “differential solubility method” (Section 3) and found out much lower yields (up to 28%), which could only be increased (up to 92%) by adding L-lysine as an additional auxiliary compound. It was supposed that, in the absence of L-lysine, the supercritical treatment succeeded in only partial complexation of the drug while the remaining molecules were simply amorphized [38].

Preliminary dissolution studies of the ketoprofen/βCD/water formulations obtained by SSI were only reported by one research group [39,42,43]. With respect to the unprocessed drug the scCO_2_-obtained samples did not show a significantly faster release but could achieve 100% dissolution of the initial drug amount [42] while the untreated sample only reached 80%. The previously mentioned incomplete complexation [38] could explain the failed improvement in the dissolution rate while the complete dissolution of the drug could be justified by the absence of crystalline clusters in the formulations.

Considering the above reported research on the ketoprofen/βCD system, effective drug formulations seem far to be obtained by the SSI method. On the other hand, successful results were recently achieved by means of a SAS-ASES technique [50]. Well-defined spherical microparticles were obtained and complete complexation was supported by DSC, X-ray diffraction and FTIR investigations. Dissolution tests performed in pH 2.5 aqueous solutions resulted in drug release rates up to seven times faster with respect to the unprocessed drug. These results appear quite promising in the prospect of future applications of this supercritical fluid technique to the ketoprofen/βCD system.

Literature reports [38,86] of ketoprofen complexation with chemically modified cyclodextrins by SSI can also be found. Results obtained with HPβCD were similar to those obtained with βCD [38]. The combined addition of water and L-lysine could increase the complexation yield up to 85%; in all samples the non-included drug was in its amorphous state. On the other hand, ketoprofen complexation with MβCD could be easily obtained without the use of any auxiliary agent thanks to the liquefaction of this host molecule during the supercritical treatment [86]. Tests in aqueous solutions at different pH values revealed almost instantaneous drug release of the scCO_2_-obtained complexes with MβCD, with 90% of the drug dissolved after 1 min, while the corresponding physical mixture required up to 27 min for the same amount of drug to be released [86].

#### 6.1.4. Piroxicam

Piroxicam has been the first NSAID to be complexed with βCD by means of an SSI technique [15] and investigations are reported in papers by different research groups [15,16,19,34,37].

Van Hees and coworkers [15,16,19] first reported successful complexation results of piroxicam and βCD, which could achieve yields up to 97% [19] without the addition of any auxiliary agent. On the other hand, Sauceau and coworkers [34] reported that water addition to the physical mixture before the SSI process was essential to achieve complexation. Complexation yields not higher than 20% could be obtained with a total humidity content of 27.5 wt.% and complete complexation could only be achieved by combining water with L-lysine addition. These results are in deep contrast with those previously reported by Van Hees [15,16,19] and, unfortunately, this difference was neither pointed out nor discussed by the authors.

The piroxicam/βCD system was also investigated by Grandelli and coworkers [37]. The main aim of the research was to demonstrate that by using specific cosolvents (i.e., ethanol) in combination with scCO_2_, higher melting point depression of piroxicam could be obtained. It was pointed out, in fact, that piroxicam undergoes thermal degradation in proximity of its melting point. This was observed upon heating piroxicam/physical mixtures at atmospheric pressure as well as processing the pure drug in supercritical atmospheres. The higher melting point depression of the drug achieved by adding a cosolvent with respect to pure scCO_2_ could prevent thermal degradation of the drug and allow its successful complexation with βCD to be achieved. Furthermore, the occurrence of thermal degradation could also be detected from a color change of the drug from yellow to brown.

Grandelli’s work [37] could also provide an explanation of the incongruence between the previously discussed results reported by Van Hees and Sauceau. This could be related to possible thermal degradation of piroxicam during the supercritical treatment, which could involve a reduction of the complexation efficiency. To support this hypothesis, it must be pointed out that Sauceau [34] detected both yellow clusters and brown powders in their supercritical-processed samples. Keeping in mind the yellow-to-brown color change connected to piroxicam degradation pointed out by Grandelli [37], this may be a hint of partial thermal degradation in some of the samples prepared by Sauceau’s research group. The occurrence of piroxicam thermal degradation and the corresponding yellow-to-brown color change were also observed in the supercritical processing of this drug with HPβCD [63], which could be avoided by operating below 140–150 °C. Inclusion yields up to 66% were obtained for the piroxicam/HPβCD system and these could be increased to 95 or 89–91% when PVP or L-lysine/water were added as auxiliary agents, respectively.

For sake of completeness, it must also be pointed out that thermal degradation is not the only aspect to be considered when piroxicam is processed in a supercritical atmosphere. It is, in fact, known that polymorphic transformation from the β to the α crystal form may also occur [37]. This was observed after treating the pure drug in pure scCO_2_ or in scCO_2_-cosolvent mixtures [37] but occurred also in the presence of HPβCD as far as the non-included drug was concerned [63]. However, until now, the consequences on the drug bioavailability of this polymorphic transition in the presence of cyclodextrins has not been investigated and could probably deserve deeper studies.

Considering all research previously discussed, it can be concluded that piroxicam complexation both with βCD and HPβCD is possible. However, much care must be taken to avoid its thermal degradation, which is the main drawback of employing this active compound. This can be achieved by employing a cosolvent to depress the melting point of the drug or by adding specific auxiliary agents such as water, L-lysine or PVP. It was also found that when complete complexation of piroxicam and βCD was achieved instantaneous dissolution of the drug in water was obtained [34]. Even though these release results can be considered very promising for pharmaceutical applications, recent reports on piroxicam SFC cannot be found. However, it would be worth performing further research on piroxicam/cyclodextrin systems, especially by investigating if antisolvent techniques could help avoid the thermal degradation’s drawback.

#### 6.1.5. Other NSAIDs

This section groups the literature reports of SFC of other NSAIDs with cyclodextrins. These drugs were less investigated with respect to those previously mentioned; however, some promising results have recently been obtained, especially as far as nimesulide is concerned [50].

TAβCD was complexed with flufenamic acid as a model drug by SSI to investigate the possibility of using this scCO_2_-soluble cyclodextrin as a carrier for sustained release [97,98]. High pressure NMR studies conducted at 40 °C and 20 MPa [97] revealed that non-inclusion complexes were formed due to the self-closure of the cyclodextrin cavity in the supercritical medium (Section 4.4). For this reason, the same research group explored the possibility of achieving inclusion complexation by processing the drug with TAβCD in its melted state since this could avoid the cavity self-closure drawback [98]. The solid-liquid-gas equilibrium of TAβCD under CO_2_ was investigated, and it was found that the cyclodextrin was melted at 35 °C and 25 MPa. When the drug and the cyclodextrin were processed under these working conditions successful complexation and sustained release results were obtained. However, the authors did not investigate if an inclusion complex had really been formed [98].

Indomethacin was processed either with HPβCD [58] and MβCD [89] by SSI. Successful complexation results were obtained with HPβCD [58], which resulted in two to three times faster release rates of the drug with respect to the corresponding physical mixtures and unprocessed indomethacin. Complexation of indomethacin with MβCD was also successful, which was supported by the absence of crystalline drug in the samples and by molecular docking studies [89]. Fast drug release rates were obtained, which were comparable to those obtained with samples prepared through spray drying.

Preliminary SSI reports of naproxen complexation with βCD [17,18] and the scCO_2_-soluble TMβCD [90] can be found in the literature. As far as βCD is concerned ethanol was employed as a cosolvent (2.5%) and both DSC scans and FTIR spectra revealed the absence of crystalline drug in the obtained samples as well as in those prepared with conventional methods such as freeze drying and spray drying [18]. In the naproxen/TMβCD system, complexation was assessed thanks to the appearance of a new crystalline phase in the X-ray spectra [90]. However, neither the naproxen/βCD or the naproxen/TMβCD formulations were subjected to dissolution investigations. The SAS-ASES technique was also employed to process naproxen either with MβCD and HPβCD [68]. Even though spherical microparticles smaller than 3 μm could be obtained for both systems, the authors evidenced that the antisolvent precipitation of MβCD could be technically hindered by its too high solubility in ethanol/scCO_2_ mixtures.

Nimesulide was processed with βCD by means of two different techniques: SSI [21] and SAS-ASES [50]. Even though an enhancement of the drug dissolution rate with respect to the corresponding physical mixtures was obtained, the SSI approach failed in achieving complete drug complexation [21]. On the other hand, the SAS-ASES technique recently allowed complete complexation and drug release rates up to 21 times faster than the unprocessed drug to be obtained [50], which would deserve further investigation, such as in vivo studies, to be performed.

### 6.2. Antifungal Drugs

Poorly-water soluble antifungal drugs were also processed with scCO_2_ to achieve their complexation with different cyclodextrins. Table 3 reports a summary of the main research activities conducted with this class of drugs. The referenced works can be divided into two sections, which mainly correspond to the research conducted by two groups by SSI. A first research group [19,22,23,35,81,82] concentrated in discussing the complexation performance of miconazole with different cyclodextrins in the presence of various acidic auxiliary agents while the second [14,24,25,33,36] investigated the complexation of itraconazole, econazole and fluconazole with βCD. However, one example of itraconazole/cyclodextrin processing with an antisolvent technique was also reported by a third group of researchers [11] and included in the second section.

#### 6.2.1. Miconazole and Miconazole Nitrate

SSI complexation of miconazole and miconazole nitrate with different cyclodextrins (βCD, HPβCD, γCD, HPγCD) in the presence of different auxiliary agents (citric, fumaric, tartaric, malic, maleic acid) was investigated by Barillaro and coworkers [19,22,81]. Successful results were obtained for many of the investigated systems. Specific studies also revealed a long-term stability of the complexes up to one year [22]. The role of different parameters such as the nature of the drug and the size of the cyclodextrin cavity were discussed to explain the complexation results of the different investigated systems. Furthermore, the role of the addition of the different auxiliary agents was extensively studied [23,35,82] to elucidate their role on the SFC mechanism.

The best complexation results were obtained for the miconazole/HPγCD/tartaric acid system, which achieved a 92% yield [22]. This system was subjected to specific in vitro and in vivo studies where the scCO_2_-obtained complexes were compared to the corresponding physical mixtures and unprocessed drug [81]. Tests resulted in improved dissolution properties and higher relative bioavailability of the supercritical-prepared samples with a 6-fold increase in C_max_ and a halved T_max_ with respect to the unprocessed drug [81].

#### 6.2.2. Itraconazole, Econazole and Fluconazole

Al-Marzouqi’s research group first processed itraconazole with different cyclodextins (αCD, βCD, γCD, HPβCD) trough SSI in a screening test [14]. Complexation with αCD and γCD could not be obtained due to the scarce compatibility of the drug with the internal cavity of these two cyclodextrins, which are probably too small to allow penetration or too large to allow a close fit of the drug, respectively [14]. Research was carried on with the itraconazole/βCD system, which exhibited the highest inclusion yields (up to 33%, evaluated with the “differential solubility method”) [24,25]. Even though the obtained complexation efficiency of the itraconazole/βCD system was not too high, in vitro and in vivo tests were performed [25]. All tests were performed with the scCO_2_-obtained complexes as well as the corresponding physical mixtures and samples prepared with a coprecipitation method. Both in vitro and in vivo studies agreed with the fact that the samples obtained by SSI gave higher dissolution amounts but not significantly faster release. The supercritical treatment, then, succeeded in increasing the drug concentration in the blood but not in accelerating its availability.

Due to the low inclusion yields obtained for the itraconazole/βCD system, the possibility of complexing βCD with lower molecular weight antifungal drugs, such as econazole and fluconazole was explored [33,36]. While incomplete inclusion or amorphization was observed for itraconazole, which displays the largest molecular structure and lowest solubility in scCO_2_, no drug crystals were detected in the econazole/βCD samples processed at the highest temperature (130 °C) or in all tested fluconazole/βCD samples. The best results obtained with fluconazole were explained with the highest solubility of this drug in scCO_2_ and the presence of two pyrrole rings in its molecular structure, which could had given rise to stronger interactions with the cyclodextrin [36]. Despite these interesting results, in vitro and in vivo studies with econazole and fluconazole have not been reported until now.

Another research group [11] reported complexation experiments of itraconazole with HPβCD by means of the SAS-ASES technique. The antisolvent technique resulted in fine (100–500 μm) amorphous particles. Even though the authors claimed that the drug was included in the cyclodextrin, no specific investigations such as NMR or FTIR spectroscopy were performed to ascertain this assumption. However, dissolution studies in an enzyme-free simulated gastric fluid resulted in enhanced release of the drug. While only 0.7% of the unprocessed drug dissolved in 120 min, up to 90% of the drug dissolved in 5–10 min from the supercritical-prepared samples. Unfortunately, this drug/cyclodextrin system has not been investigated further.

### 6.3. Essential Oils and Other Natural Compounds

Many natural products or extracts from plants such as essential oils, alkaloids, carotenoids, flavonoids, or other phenolic compounds possess antioxidant, anti-inflammatory, antimicrobial and anticancer properties. For these reasons they are being used in pharmaceutical formulations due to their beneficial effects on human health [105]. Many of these compounds are insoluble or poorly soluble in water and, for this reason, their complexation with cyclodextrins can increase their bioavailability. Furthermore, their solubility in scCO_2_ has made their recovery through supercritical fluid extraction from herbs quite popular in the last years [5]. It is not surprising, then, that many researchers have attempted their encapsulation in cyclodextrins through supercritical fluid technologies. The main SFC results conducted with natural compounds are summarized in Table 4 and discussed in the following sections. The first section focusses on essential oils, which are volatile natural compounds while the second section discusses the other non-volatile plant extracts with medicinal properties.

#### 6.3.1. Essential Oils

The curative effect of essential oils may be negatively affected by their instability since they tend to either volatilize or oxidize [44]. For this reason, as well as to increase their bioavailability, essential oils are often complexed with cyclodextrins and the SSI approach has often been employed to achieve this goal [20,41,44,45,61,67,85].

Locci and coworker [20] were probably the first researchers to investigate the SFC of essential oils with a cyclodextrin. They investigated the interactions of carvacrol, thymol and eugenol with βCD to provide evidence of the effective formation of inclusion complexes with NMR techniques. Thymol [67] and carvacrol [45] were further investigated by other research groups, who also compared their encapsulation efficiency into cyclodextrin cavities with those obtained by conventional methods. Even though successful complexation of thymol with HPβCD was obtained by Pires and coworkers [67], better complexation efficiency, drug bioavailability and thermal stability were obtained with a conventional freeze drying method. Similarly, Al-Shar’i and coworkers [45], who investigated the SFC of carvacrol and linalool with βCD, pointed out that a conventional kneading method provided more efficient molecular entrapment in the cyclodextrin cavity.

Safranal [41] and menthol [44] were also complexed with βCD by SSI. Complexation yields up to 48.5% were obtained with the safranal/βCD system, which decreased only to 41.2% after one-year storage due to safranal volatilization. Drug dissolution studies showed that the complexes obtained with sealed-heating and SSI had faster release rates than pure safranal as well as the complexes obtained with other conventional methods [41]. On the other hand, menthol complexation with βCD required the presence of a cosolvent [44]. A maximum inclusion yield of 93.7% was obtained by treating the components in a scCO_2_-water (5%) mixture. In vitro release studies in a pH1 solution revealed a fast dissolution rate of the obtained complexes with 97.8% of menthol released after 1.5 h, which was much higher than that released by the untreated menthol (11.2%) after the same time [44].

The melting point depression of MβCD in scCO_2_ was exploited to investigate its complexation with borneol, cinnamaldehyde and muscone [61,85]. While incomplete complexation of muscone with MβCD was obtained, which was due to the too large size of the drug molecule with respect to the cyclodextrin cavity, complete complexation was achieved both with borneol and cinnamaldehyde. The authors did not perform in vitro release tests but measured the apparent solubility of the complexed drugs in water. Thanks to SFC, this was increased about 70 times for borneol [61] and about 20 times for cinnamaldehyde [85], respectively. Furthermore, the formulations obtained by SSI exhibited much higher thermal stability with respect to those prepared with a conventional sealed heating approach. Thermal gravimetric analyses revealed a 50% mass loss of borneol from the samples obtained with the sealed-heating method between 125 and 185 °C while only 10% of the drug was vaporized from SSI formulations when heated between 150 and 190 °C [61]. As far as cinnamaldehyde is concerned, thermostability of the formulations was tested by thermostat oven heating at 60 °C. While the samples prepared by sealed heating displayed more than 10% mass loss of cinnamaldehyde after 6 h, no loss over 6 days was observed for those obtained by SSI [85] This was explained by stronger interactions between each drug and the cyclodextrin when their complexation occurred in the supercritical medium. DSC and X-ray analyses pointed out the formation of a more stable crystalline complex of borneol with MβCD after the supercritical treatment, which was not found after the sealed heating treatment [61].

The above overview has pointed out that the supercritical fluid technology can be successfully used to complex essential oils with cyclodextrins even though the results in terms of improved bioavailability and thermal stability are not often better than those of the conventional methods. A preliminary observation of the results points out that the most effective stabilization of these volatile compounds can be probably obtained by using MβCD, which may increase host/guest interactions thanks to the direct dissolution of the drug in the melted cyclodextrin, which occurs in the presence of scCO_2_ (Section 4.1).

#### 6.3.2. Non-Volatile Natural Compounds

This section summarizes the main research conducted with non-volatile natural active compounds, such as alkaloids, carotenoids, flavonoids, or other phenolic compounds with different supercritical fluid techniques. Successful results were obtained in many of the investigated systems, especially as far as the use of the antisolvent approaches is concerned, which were also supported by promising in vitro investigations.

The difference in the complexation performance by SSI of anisole, asarone, curcumin and shikonin, which are natural plant extracts commonly employed in Traditional Chinese Medicine, either with MβCD or HPβCD was investigated [59,62]. All drugs could much more easily complex with MβCD, thanks to the enhanced interaction efficiency connected with the liquefaction of this cyclodextrin in scCO_2_ (Section 4.1). The improvement in aqueous solubility was only tested for shikonin, which exhibited a 75% enhancement thanks to its SFC with MβCD [59]. Complexation efficiency with both cyclodextrins was related to the molecular structure and the drug solubility in the supercritical medium. Since curcumin molecule has a more complex structure and exhibits the lowest solubility in scCO_2_ its complexation with MβCD required higher temperatures and longer process time [62]. Instead, another research group [73,74] succeeded in processing curcumin with HPβCD and PVP by means of the antisolvent ARISE technique. It was found that coprocessing curcumin with both excipients resulted in the reduction of the drug crystallinity with a consequent increase of its solubility in aqueous solutions up to 70 times [74]. Furthermore, light inhalable powders with enhanced aerodynamic properties (i.e., inhalable fraction up to 61%) [74] were obtained and in vitro cytotoxicity tests revealed an improved drug biodistribution and release in lung cancer cells [73].

Phenolic and polyphenolic compounds are the most investigated class of non-volatile herbal ingredients as far as SFC is concerned. The SSI approach has recently been attempted with daidzein, catechin and baicalin. Daidzein complexation with HPβCD was compared with a conventional lyophilization process [64]. Even though the authors claimed that inclusion yields of the scCO_2_-obtained complexes were higher, the reported in vitro dissolution tests displayed much faster drug release for the lyophilized samples. The catechin/βCD system was processed through SFC and conventional techniques [40]. Different analytical techniques supported the formation of an inclusion complex with all approaches and the measurement of the catechin antioxidant activity pointed out that this was four times higher than that of the unprocessed drug, irrespective of the adopted complexation method. Baicalin was complexed with HPβCD [66] and, even though the inclusion efficiency was quite low, the obtained complexes exhibited an improved apparent aqueous solubility (up to 1.3 mg/mL) higher than the untreated baicalin (0.08 mg/mL) and lyophilized samples (0.3 mg/mL). This was ascribed to the reduced particle size and the absence of drug crystals in the supercritical formulations. The addition of L-lysine allowed the inclusion efficiency of baicalin to be increased more than six times, however it was not reported at what extent this could affect the apparent water solubility of the drug [66].

The complexation of phenolic compounds with antisolvent techniques was also investigated. With respect to the SSI approach, the SAS methods seem to lead to more promising results, probably thanks to the possibility of matching drug complexation with particle size reduction. For example, the SAS-SEDS processing of resveratrol with HPβCD resulted in amorphous particles with enhanced water solubility and dissolution rate [70]. While the unprocessed drug was practically insoluble in water solutions, the obtained formulations allowed an apparent solubility of 25 mg/mL to be obtained. In vitro tests pointed out that 100% of the drug could be dissolved in 5–10 min with a release rate two times faster than the corresponding physical mixtures [70]. Complete or almost complete complexation yield was also obtained by processing the puerin/βCD system by means of a SAS-SEDS method [48]. Drug release tests were conducted on the obtained nanoparticles and revealed an improved dissolution rate with 98.6% of the drug released after 5 min, which was higher than the 68.7 and 78.7% released from the untreated drug and the physical mixtures [48]. However, the most complete and promising results were obtained with apigenin [72] and baicalein [80]. Successful complexation (up to 93%) of apigenin with HPβCD was obtained with a SAS-ASES process [72]. Nanometric particles were obtained, which resulted in an apparent aqueous solubility of the drug more than 150 times higher than that of the unprocessed drug. In vitro and in vivo studies of the complexes resulted in apigenin dissolution rate and bioavailability more than six times higher than those of the unprocessed drug [72]. Recently, Baicalein complexation with HPβCD by using a SAS PCA/ASES technique [80] has resulted in nanometric amorphous particles with enhanced aqueous solubility (up to 10 times higher than the unprocessed drug). In vitro and in vivo studies were also performed. The bioavailability of baicalein increased up to eight times while the antibacterial ant antioxidant activities were also improved [80].

The complexation of other natural nonphenolic extracts has also been investigated with SAS-SEDS. Lycopene, which is a carotenoid with antioxidant activity, was processed with βCD at different working conditions. Amorphous particles whose minimum particle size was equal to 38 nm were obtained while in vitro and in vivo studies have not been performed yet [47]. Berberine, a natural alkaloid with many pharmacological properties, has recently been complexed with βCD [49]. Its complexation was confirmed by NMR studies, and it was found that the working conditions of the process could strongly influence the particle size of the complex, which could range from the micro to the nanoscale. In vitro dissolution studies pointed out an improvement of the release rate especially of the nano-size particles. In vivo pharmacokinetic parameters also revealed improvement of the drug bioavailability: C_max_ increased from 25.5 μg/mL of the raw drug to 33.1 and 54.5 μg/mL of the micro and nano complexes, respectively, while T_max_ decreased from 1 to 0.85 and 0.75 h, respectively (49).

Until now the SAA/SASD particle formation technique, where scCO_2_ acts as a co-solute, has only been used to process propolis extracts with HPβCD [77]. Propolis is a natural resinous mixture rich in bioactive compounds. Pharmaceutical applications of propolis extracts are limited by their chemical and enzymatic degradation, which can be overcome by their encapsulation in cyclodextrins [77]. In this context, SAA/SASD resulted in the production of submicrometric spherical particles in which propolis extracts and HPβCD were coprecipitated. The particles contained up to 100% of the total polyphenol content that was present in the initial liquid solution before micronization. Furthermore, the measurement of the radical scavenging activity demonstrated that the antioxidant properties of propolis extracts were preserved from degradation thanks to the interaction with the carrier [77].

### 6.4. Other Drugs Processed with SFC Technologies

Table 5 summarizes research conducted on drugs that were not included in previous sections. The table is an interesting overview of the wide spectrum of different classes of drugs that were tested over the last years with supercritical fluid techniques. Remarkable drug release improvement was found in most cases and promising in vivo studies were also obtained for pharmaceutical formulations of some drugs, such as carbamazepine [75], dutasteride [71] and simvastatin [69] as well as the achievement of an effective taste masking for the cetirizine/βCD complexes [46]. The main results obtained for each drug are discussed in the following paragraphs.

#### 6.4.1. Albendazole

Albendazole/βCD microparticles, with a mean diameter ranging from 0.45 to 1.4 μm, were formed with a SAS-SEDS approach [51]. The characterization techniques evidenced the presence of a new solid phase, which was attributed to inclusion complex formation. Furthermore, thanks to the supercritical treatment, the non-included drug underwent a polymorphic transition from tautomeric form I to form II. In vitro release tests showed that the albendazole/βCD particles exhibited a drug release rate 3.5 faster with respect to those of the pure albendazole obtained by the same SEDS process and a physical mixture with the same molar ratio. Even though the tautomeric form II of albendazole generally exhibits improved dissolution performances, the authors concluded that this was not enough to justify the huge increase in the dissolution rate of the scCO_2_-obtained samples, which was instead attributed to the formation of an inclusion complex [51].

#### 6.4.2. Benznidazole

Benznidazole is an antiparasitic drug employed in the treatment of Chagas disease. The SSI and freeze-drying complexation of this drug with γCD have recently been compared [53]. Samples were characterized with different techniques. While the scCO_2_-obtained samples displayed higher complexation yield (54% vs. 11%) those obtained with freeze drying displayed higher amorphization of the drug, which resulted in much faster release rate of these last formulations during in vitro dissolution tests.

#### 6.4.3. Benzocaine, Bupivacaine, Mepivacaine

The local anesthetic agents, benzocaine, bupivacaine and mepivacaine were processed with βCD by SSI and their results were compared with those obtained with different conventional techniques [30,31]. The samples obtained with the co-grinding technique were the only ones where drug crystals were not detected. Dissolution studies indicated that all techniques were more effective than the simple physical mixtures in improving the release of the drugs. However, no general rule to select the most effective preparation method could be proposed since the release rank orders differed for each drug/cyclodextrin system [31].

#### 6.4.4. Budesonide

Budesonide, a corticosteroid drug, was processed either with HPβCD [58] and γCD [54,55] through two different techniques. Complexation with HPβCD was achieved by SSI [58] which led to successful results and release rates (87% of drug release after 45 min) faster than those of the physical mixtures (53% after 45 min) and the unprocessed drug (14% after 15 min). Complexation with γCD [54,55] was instead performed with SEDS. The release rate of the complexed budesonide (up to 93% after 1 min) was faster than that obtained by a conventional coprecipitation method (62% after 1 min). This was explained by the tetragonal-channel structure of the γCD in the budesonide/γCD complexes, which was promoted by the supercritical treatment. In the samples obtained with the conventional coprecipitation technique, instead, γCD exhibited a hexagonal-channel pattern. When γCD in the complexes exhibited a tetragonal-channel form, release test showed faster dissolution rates with respect to those where the cyclodextrin exhibited a hexagonal-channel one. The tetragonal-channel form, in fact, displays lower tightness of the molecules in the unit cell, which could facilitate water molecules penetration [55].

#### 6.4.5. Carbamazepine

Carbamazepine, an antiepileptic drug, was complexed with γCD by means of a SAS-GAS approach [56]. The binary complexes were compared to ternary ones where nicotinamide was employed as an auxiliary agent. However, nicotinamide was not simply added to the drug/cyclodextrin physical mixtures before the SAS treatment. Co-crystals of carbamazepine and nicotinamide were first precipitated from an ethanolic solution with GAS, then the obtained co-crystals were complexed with γCD through another subsequent GAS step. According to the authors [56] the co-crystals may provide significant increase in the bioavailability of this poorly-water soluble drug. X-ray spectra and DSC scans confirmed the formation of the carbamazepine/nicotinamide co-crystals as well as the formation of amorphous formulations with the cyclodextrin. The intrinsic dissolution rate constants of the different formulations were measured and compared. The carbamazepine/nicotinamide co-crystals resulted in a 2.5-fold increase in the dissolution rate with respect to the pure drug; the binary carbamazepine/γCD systems provided an 8-fold increase while the ternary carbamazepine/nicotinamide/γCD ones were able to enhance the release up to 30–40 times [56].

The efficacy of the obtained ternary formulations was evaluated by measuring dopamine responses with in vivo brain microdialysis after oral administration [75]. Compared to the pure drug, the ternary formulations produced elevated levels of dopamine (up to 250% of the baseline) with a stepwise increase and a peak of concentration after 1.5–2 h. According to the authors the presence of nicotinamide does not affect the drug absorption in the gastrointestinal tract after release, since the two components in the co-crystals tend to dissociate after the dissolution [75].

#### 6.4.6. Captopril, Molsidomine, Omeprazole

Captopril, molsidomine and omeprazole were complexed with sc-CO_2_ soluble cyclodextrins by SSI to achieve sustained release [91,92,93,94,97]. In vitro release tests were conducted in oily suspensions [91,92,93,94] where the oil serves as a vehicle for sustained release preparations since sc-CO_2_ soluble cyclodextrins are not water soluble.

Captopril SFC either with PAβCD [93] or TAβCD [97] was investigated. While the captopril/TAβCD system was mainly studied to elucidate the inclusion or non-inclusion nature of complexation with high pressure NMR studies [97], the captopril/PAβCD was also subjected to in vitro dissolution tests, which revealed the successful attainment of a sustained release of this drug [93]. While drug dissolution of the physical mixtures and unprocessed captopril was complete after 0.5 h, the released percentage from the supercritical samples after 2 h was equal to 60% and slowly reached the maximum after 6 h [93].

A fluorinated cyclodextrin, FAγCD, was synthesized [91] as a potential sustained release carrier for molsidomine, a vasodilating drug. The unprocessed molsidomine and its physical mixtures with the cyclodextrin displayed a very fast release while only 45% of the drug dissolved after 3 h from the scCO_2_-obtained complex; the release rate was almost linear and reached complete drug dissolution after 8.5 h [91]. The same drug was processed with PAβCD and similar results were obtained: complete dissolution was slowly achieved after 7.5 h [92].

Omeprazole was also successfully complexed with PAβCD and resulted in remarkable delay of drug release. While the unprocessed drug and physical mixtures released 100% of omeprazole after 2 h, the scCO_2_-obtained complexes only released 65% of the drug after the same time, which slowly increased to 82% after 4 h and reached complete dissolution after 7.5 h [94].

#### 6.4.7. Cetirizine Hydrochloride

Cetirizine hydrochloride is an antihistaminic drug with a very unpleasant bitter taste. The encapsulation into the cavity of a cyclodextrin may help mask the unpleasant taste of cetirizine since this prevents the drug to be attached to the taste-bud receptors in the mouth cavity. This is the main reason why cetirizine was complexed with βCD by means of SAS-ASES [46]. The antisolvent approach was compared with a conventional freeze- drying method. Taste perception tests over a panel of fifteen volunteers showed that both techniques succeeded in effective taste masking, which did not happen with the simple drug/cyclodextrin physical mixing. The best evaluation scores were obtained with the ASES-prepared formulations probably because this technique resulted in complete drug encapsulation as it was evidenced by different analytical techniques [46].

#### 6.4.8. Dutasteride

Dutasteride was processed with HPβCD with a SAS-ASES preparation method in the presence of different hydrophilic auxiliary agents, such as hydroxypropyl cellulose (HPC), hydroxypropylmethyl cellulose (HPMC), polyvinyl pyrrolidone (PVP), polyvinyl pyrrolidone- vinyl acetate (PVP-VA), polyethylene glycol (PEG), poloxamer, ryotoester [71]. Nanoparticle amorphous aggregates were obtained and were subjected to supersaturation, dissolution rate and in vivo studies. If was found that in vivo pharmacokinetic parameters were more closely correlated with those of the supersaturation studies than those of the dissolution rate investigations. It was concluded that to achieve higher bioavailability it was more important prolonging supersaturation than improving the dissolution rate. Among the different formulations, the dutasteride/HPβCD nanostructures with HPMC exhibited the best bioavailability that was comparable to that of a commercially available soft gelatin capsule of the same drug [71].

#### 6.4.9. Eflucimibe

Eflucimibe is a drug that displays hypocholesterolemic properties and was processed with γCD by Rodier and coworkers [52]. The authors proposed a multi-step technique that matched the supercritical antisolvent co-precipitation of the drug and the cyclodextrin from a DMSO solution with the SSI of the coprecipitated components in static mode in the presence of water, which was added as an auxiliary agent. This resulted in almost complete inclusion or amorphization of the drug and improved dissolution properties. In fact, the concentration of the drug dissolved in sodium-dodecyl-sulfate, which was the release medium, after 2 h was 35 times higher than that observed for the drug/cyclodextrin physical mixtures. The benefit of matching the coprecipitation step with SSI treatment was demonstrated by the fact that the simple SSI treatment of a physical mixture only led to a 7.5 times improvement in the released drug concentration during the release tests.

#### 6.4.10. Ibersartan

A SAS-ASES micronization technique has recently been used to increase the aqueous solubility and dissolution rate of poorly-water soluble drug, ibersartan [76]. The same antisolvent technique was used to prepare ibersartan microparticles as well as its formulations with different carriers, such as PVP and HPβCD. All samples resulted in improved aqueous solubility and drug dissolution rate. The formulations with PVP and HPβCD displayed an amorphous state and resulted in faster drug release (90% of the drug released after 20 min, with respect to the 35% of the untreated drug dissolved after 120 min).

#### 6.4.11. Lopinavir

Lopinavir is an antiretroviral drug with limited oral bioavailability that can also be used for the clinical management of HIV infections. Its complexation with different cyclodextrins has recently been investigated both with molecular dynamic simulations, SASD and coevaporation [57]. According to the simulations the best host-guest molecular interaction should be achieved with a hydroxypropyl-γ-derivative with high degree of substitution (HP17γCD). The HP17γCD was synthesized and compared with γCD and commercial HPγCD as far as its experimental complexation with lopinavir via SASD or coevaporation were concerned. Results indicated a higher drug amorphization and solubilization ability of HP17γCD with both techniques, which confirmed the molecular dynamics simulation results. The SASD processing technology resulted in higher drug amorphization and release from complexes. Even though the burst effect typically observed for cyclodextrin complexes was not observed, the drug/HP17γCD formulations prepared with SASD showed the highest amount of drug dissolved in the first 60 min (51.7%) with respect to the negligible amount released from the unprocessed drug [57].

#### 6.4.12. Olanzepine

The melting point depression of MβCD favored by scCO_2_ was exploited to encapsulate the neuroleptic drug olanzapine by SSI [88]. Complete amorphization or inclusion of the drug was suggested by different analytical tests as well as molecular computation models. Dissolution studies of the samples resulted in very fast release with 90% of the drug dissolved after 10 min with respect to the 17% released by the unprocessed drug or the physical mixtures after 60 min. The scCO_2_-obtained complexes exhibited significant faster release rate also with respect to samples obtained with co-evaporation and freeze- drying methods, which did not exhibit complete drug inclusion or amorphization.

#### 6.4.13. Simvastatin

Simvastatin is a lipid-lowering drug that is practically insoluble in water and was processed with HPβCD by means of SAS-SEDS to increase its bioavailability [69]. Amorphous formulations with enhanced aqueous solubility (more than 9.5-fold higher than the unprocessed drug) were obtained. Dissolution rate studies also revealed remarkable improvement with 73% of simvastatin released after 10 min, which was higher than the 4% and 49% dissolved from the unprocessed drug samples and their physical mixture with HPβCD after the same time. In vivo studies in rats were performed to test the hypolipidemic activity of the formulations. The animals were administered an excess of coconut oil to promote hypercholesterolemia. While a group received an aqueous suspension containing simvastatin, another group received a suspension with the drug/cyclodextrin formulation. It was found that the SEDS-prepared formulation performed better than simvastatin in reducing total cholesterol and triglyceride levels [69].

#### 6.4.14. Tosufloxacin Tosylate

The complexation of the antibiotic tosufloxacin tosylate with HPβCD was investigated by the same research group with two different versions of the antisolvent approach: the GAS [78] and the SEDS [79]. In both works the authors investigated different temperature and pressure ranges, which resulted in slightly different optimal working conditions (45°C and 12 MPa with GAS; 35°C 16 MPa with SEDS). The main difference consisted in the particle size. A minimum average diameter of 28.56 μm was obtained with GAS (78) while smaller spherical 1.91 μm particles could be obtained with SEDS [79], which is due to the higher mixing efficiency of the SEDS coaxial nozzle (Section 4.1). The GAS-prepared formulations resulted in a maximum complexation yield of 29% [78] while NMR and molecular modeling studies indicated that tosufloxacin tosylate was totally embedded into the cavity of HPβCD of the SEDS-prepared formulations [79]. The drug dissolution studies revealed in both cases a significant increase of the drug release rate (3.5–5 times faster) with an apparent water solubility 6.6 times higher than that of the unprocessed drug [79], which was ascribed more to the reduced particle size than the complexation efficiency [78]. In vitro tests showed that the antibacterial activity of tosufloxacin tosylate was maintained after the SFC [79] while in vivo experiments have not been performed yet.

## 7. Conclusions

The extensive research conducted over the last decades points out that achieving drug/cyclodextrin complexation through supercritical fluid technology as a green alternative process is possible. Different complexation techniques are available, whose mechanisms were discussed and at least partially elucidated. Table 6 reports a brief summary of the supercritical fluid complexation technologies, their advantages and disadvantages. For sake of completeness, the table also reports some information about the conventional complexation methods employed as a reference in the papers here reviewed. The supercritical preparation method is applicable to a wide variety of different drug classes. However, despite the fact that over 50 different drugs have been tested, only few of them resulted in pharmaceutical preparations that underwent both in vitro and in vivo studies [49,65,69,71,72,75,80,81] and, to the author’s knowledge none of them has never been included in commercial formulations until now. This could be related to the complexity and high costs of the high-pressure equipment required by the supercritical fluid techniques. Even though a direct comparison of the cost-benefit ratio between conventional and supercritical-mediated complexation techniques is still missing in the literature, probably, the “green” benefit of scCO_2_ is not enough to justify its employment from an economic point of view. When compared to conventional complexation techniques, the scCO_2_-obtained formulations often resulted in comparable performances and only few cases resulted in remarkable improvement [46,54,55,61,85,88].

Future research efforts should be concentrated in exploiting the properties of scCO_2_ in order to “make the difference” not only from an environmental point of view but also as far as the properties of the obtained formulations are concerned. It was shown [59,62,84,85,86,88], for example, how scCO_2_ has the peculiarity of favoring melting point depression of MβCD, which does not occur with other preparation methods. Another feature of the supercritical medium is the possibility of processing scCO_2_-soluble cyclodextrins to achieve sustained release formulations [91,92,93,94]. Furthermore, the SFC with both MβCD and scCO_2_-soluble cyclodextrins can be achieved by SSI, which is the simplest technological approach. Future research could be aimed at broadening the complexation of other drugs with these types of cyclodextrins as well as performing in vitro studies, which appear to be completely missing now. As far as scCO_2_-soluble cyclodextrins are concerned, particle-formation techniques where scCO_2_ acts as a solvent, such as the rapid expansion of supercritical solutions (RESS) process could also be investigated. To the author’s knowledge, in fact, RESS has not been employed as a complexation technique yet, even though it could match the advantages of the SSI approach for scCO_2_-soluble cyclodextrins with the possibility of tuning their particle size upon depressurization.

Even though the SAS techniques (GAS, SEDS, ASES) are much complex to design, they have already resulted in many of the most promising results [46,49,69,71,72,75] thanks to the possibility of matching drug/cyclodextrin complexation with particle size reduction. The same advantage can also be achieved with the SAA/SASD approach where the scCO_2_ acts as a pneumatic agent. In the author’s opinion the SAS and SAA/SASD are the most innovative and versatile technologies to be explored but specific investigation should be performed to understand how they can affect not only the particle size and morphology but also the inclusion complexation efficiency. Furthermore, in vitro and in vivo experiments with complexes obtained at different particle sizes could also be conducted to investigate how this may affect the drug bioavailability. All these aspects could effectively help better design the drug release profile of the formulations.

Eventually innovation in supercritical fluid technology should try not only to mimic the conventional complexation processes but propose new routes for the preparation of novel pharmaceutical carriers. A recent example is the supercritical preparation and drug loading of cyclodextrin-based highly porous metal-organic frameworks [109]. These are highly porous materials that can be effectively employed as drug carriers. The use of scCO_2_ allows carriers with higher loading capacity to be obtained since it avoids the collapse of the porous structure. On the other hand, the higher diffusivity and penetration capacity of the scCO_2_ allows more efficient host-guest encapsulation of the drugs as well as their penetration in the confined spherical cavities of the carrier to be obtained.

## Figures and Tables

**Table 1:** Cyclodextrins employed in SFC studies.

	Cyclodextrin	Acronym	Ref.
ScCO_2_-insoluble	α-cyclodextrin	αCD	[14]
	β-cyclodextrin	βCD	[14,15,16,17,18,19,20,21,22,23,24,25,26,27,28,29,30,31,32,33,34,35,36,37,38,39,40,41,42,43,44,45,46,47,48,49,50,51]
	γ-cyclodextrin	γCD	[14,19,22,23,52,53,54,55,56,57]
	hydroxypropyl-β-cyclodextrin	HPβCD	[11,14,19,22,23,38,58,59,60,61,62,63,64,65,66,67,68,69,70,71,72,73,74,75,76,77,78,79,80]
	hydroxypropyl-γ-cyclodextrin	HPγCD	[19,22,23,57,81,82,83]
	methyl-β-cyclodextrin	βCD	[59,61,62,68,84,85,86,87,88,89]
ScCO_2_-soluble	dimethyl-β-cyclodextrin	DMβCD	[26]
	trimethyl-β-cyclodextrin	TMβCD	[26,90]
	perfluorobutanoyl-γ-cyclodextrin	FAγCD	[91]
	peracetylated-β-cyclodextrin	PAβCD	[92,93,94,95,96]
	triacetyl-β-cyclodextrin	TAβCD	[97,98]

**Table 2:** NSAID drugs employed in the SFC with cyclodextrins.

Drug	Cyclodextrin	Technique	Temperature and Pressure	Solvent or Cosolvent	Auxiliary Agents	Ref.
Flufenamic acid	TAβCD	SSI	35–40 °C 20–25 MPa			[97,98]
Flurbiprofen	TMβCD	SSI	35 °C 12 MPa			[90]
	MβCD	SSI	35–45 °C 10–20 MPa			[87]
	HPβCD	SSI	60 °C 26 MPa			[65]
Ibuprofen	βCD, DMβCD, TMβCD	SSI	35 °C 12 MPa			[26,90]
	βCD	SSI	40 °C 25–30 MPa			[27,28]
	MβCD	SSI	35 °C 13–22 MPa			[84]
	HPγCD	SAA/SASD	65 °C 12.8 MPa	ethanol, water		[83]
	PAβCD	SSI	35 °C 25 MPa			[96]
	βCD granules	SSI	40 °C 25 MPa			[29]
	PMMA functionalized with HPβCD	SSI	40 °C 20 MPa			[60]
Indomethacin	HPβCD	SSI	40 °C 21 MPa			[58]
	MβCD	SSI	35–45 °C 10–20 MPa			[89]
Ketoprofen	βCD	SSI	65–75 °C 15–20 MPa		water	[32]
	βCD	SSI	50 °C 8 MPa		water	[43]
	βCD	SSI	30–50 °C 8–12 MPa	water		[39,42]
	βCD	SAS-ASES	40 °C 9–12 MPa	DMSO		[50]
	βCD, HPβCD	SSI	40–85° 15–30 MPa		water, L-lysine	[38]
	βCD	SSI	40 °C 20 MPa			[86]
Naproxen	βCD	SSI	62 °C 16 MPa	ethanol		[17,18]
	TMβCD	SSI	35 °C 12 MPa			[90]
	MβCD, HPβCD	SAS-ASES	25 °C 6.5–16 MPa	acetone, ethanol, DMSO		[68]
Nimesulide	βCD	SSI	40–130 °C 14–22 MPa			[21]
	βCD	SAS-ASES	40 °C 9–15 MPa	DMSO		[50]
Piroxicam	βCD	SSI	50–150 °C 15–50 MPa		L-lysine, trometamol	[15,16,19]
	βCD	SSI	110–150 °C 15 MPa		water, L-lysine	[34]
	βCD	SSI	160 °C 29 MPa	ethanol		[37]
	HPβCD	SSI	100–150 °C 30 MPa		water, L-lysine, PVP	[63]

**Table 3:** Antifungal drugs employed in the SFC with cyclodextrins.

Drug	Cyclodextrin	Technique	Temperature and Pressure	Solvent or Cosolvent	Auxiliary Agents	Ref.
Econazole	βCD	SSI	75–130 °C 10–45 MPa			[33,36]
Fluconazole	βCD	SSI	100–130 °C 10–45 MPa			[36]
Itraconazole	αCD, βCD, γCD, HPβCD	SSI	50–130 °C 25–35 MPa			[14,24,25,36]
	HPβCD	SAS-ASES	35–55 °C 8.3–14 MPa	DCM, ethanol		[11]
Miconazole	βCD, HPβCD, γCD, HPγCD	SSI	125 °C 30 MPa		citric, malic, tartaric, maleic, fumaric acid	[19,22,23,35,81,82]
Miconazole nitrate	βCD, HPβCD, γCD, HPγCD	SSI	125 °C 30 MPa		citric, malic, tartaric, maleic, fumaric acid	[19,22]

**Table 4:** Essential oils and other natural compounds employed in the SFC with cyclodextrins.

Drug	Cyclodextrin	Technique	Temperature and Pressure	Solvent or Cosolvent	Auxiliary Agents	Ref.
Anisole	MβCD, HPβCD	SSI	50–80 °C 5–7 MPa			[62]
Apigenin	HPβCD	SAS-ASES	35–65 °C 10–25 MPa	DMF		[72]
Asarone	MβCD, HPβCD	SSI	55–75 °C 5–10 MPa			[62]
Baicalein	HPβCD	SAS-PCA/ASES	35–50 °C 8–14 MPa	acetone, ethanol		[80]
Baicalin	HPβCD	SSI	45–65 °C 10–30 MPa		L-lysine	[66]
Berberine	βCD	SAS-SEDS	40 °C 9–15 MPa	DMSO, DCM		[49]
Borneol	MβCD	SSI	90–140 °C 7–20 MPa			[61]
Carvacrol	βCD	SSI	50 °C 8 MPa			[20]
	βCD	SSI	40 °C 10 MPa			[45]
Catechin	βCD	SSI	40 °C 9 MPa			[40]
Cinnamaldehyde	MβCD	SSI	50–100 °C 7–10 MPa			[85]
Curcumin	MβCD, HPβCD	SSI	100–140 °C 7–112 MPa			[62]
	HPβCD	SAS-ARISE	25–45 °C 9.5 MPa	acetone, ethanol, methanol	PVP	[73,74]
Daidzein	HPβCD	SSI	200 °C 20 MPa			[64]
Eugenol	βCD	SSI	50 °C 8 MPa			[20]
Linalool	βCD	SSI	40 °C 10 MPa			[45]
Lycopene	βCD	SAS-SEDS	40–50 °C 10–14 MPa	DMF, DMSO, DCM		[47]
Menthol	βCD	SSI	40–70 °C 10–30 MPa	ethanol, water		[44]
Muscone	MβCD	SSI	50–100 °C 7–10 MPa			[85]
Propolis	HPβCD	SAA	90 °C 9 MPa	ethanol, water		[77]
Puerarin	βCD	SAS-SEDS	35–55 °C 10–20 MPa	DMSO		[48]
Resveratrol	HPβCD	SAS-SEDS	40 °C 12 MPa	ethanol		[70]
Safranal	βCD	SSI	35–55 °C 10–30 MPa			[41]
Shikonin	MβCD, HPβCD	SSI	80–100 °C 7–15 MPa			[59]
Thymol	βCD	SSI	50 °C 8 MPa			[20]
	HPβCD	SSI	50 °C 24 MPa			[67]

**Table 5:** Other drugs employed in the SFC with cyclodextrins.

Drug	Drug Type	Cyclodextrin	Technique	Temperature and Pressure	Solvent or Cosolvent	Auxiliary Agents	Ref.
Albendazole	anthelmintic	βCD	SAS-SEDS	40 °C 9–15 MPa	acetone, DMSO		[51]
Benznidazole	antiparasitic	γCD	SSI	37–47 °C 25 MPa			[53]
Benzocaine	anesthetic	βCD	SSI	50–100 °C 10–45 MPa			[30,31]
Budesonide	corticosteroid	HPβCD	SSI	40 °C 21 MPa			[58]
		γCD	SAS-SEDS	40–80 °C 10–20 MPa	ethanol, water		[54,55]
Bupivacaine	anesthetic	βCD	SSI	50–100 °C 10–45 MPa			[31]
Captopril	ACE inhibitor	PAβCD	SSI	45 °C 34.5 MPa			[93]
		TAβCD	SSI	40 °C 20 MPa			[97]
Carbamazepine	antiepileptic	γCD	SAS-GAS	40 °C 13.5–11 MPa	ethanol	nicotinamide	[56,75]
Cetirizine hydrochloride	antihistaminic	βCD	SAS-ASES	35 °C 15 MPa	DMSO		[46]
Dutasteride	5α-reductase-inhibitor	HPβCD	SAS-ASES	40 °C 15 MPa	ethanol, DCM	HPC, HPMC, PVP, PVP-VA, PEG, poloxamer, ryotoester	[71]
Eflucimibe	Hypocholesterolemic, antiatherosclerotic	γCD	SSI	40–100 °C 10–30 MPa		water	[52]
Irbesartan	angiotensin receptor blocker	HPβCD	SAS-ASES	35–50 °C 8–16 MPa	ethanol, DMSO		[76]
Lopinavir	antiretroviral	γCD, HPγCD	SASD	65*C 10 MPa	ethanol, water		[57]
Mepivacaine	anesthetic	βCD	SSI	75–100 °C 10–45 MPa			[31]
Molsidomine	vasodilating	FAγCD, PAβCD	SSI	45 °C 34.5 MPa			[91,92]
Olanzapine	neuroleptic	MβCD	SSI	45–55 °C 12–20 MPa			[88]
Omeprazole	proton pump inhibitor	PAβCD	SSI	45 °C 34.5 MPa			[94]
Simvastatin	lipid-lowering	HPβCD	SAS-SEDS	40 °C 12 MPa	ethanol, DCM		[69]
Tosufloxacin tosylate	antibiotic	HPβCD	SAS-GAS,	35–55 °C 8–16 MPa	DMF, DCM		[78]
			SAS-SEDS	35–50 °C 8–16 MPa	DMF		[79]

**Table 6:** Brief description, advantages and disadvantages of different scCO_2_ -mediated and conventional approaches for the preparation of drug/cyclodextrin complexes.

	Complexation Technique	Advantages	Disadvantages
ScCO_2_-mediated techniques	SSI: drug and cyclodextrin are placed into a vessel and contacted with scCO_2_ at constant temperature and pressure for a fixed period.	absence of residual solvent;	no control of particle size;
		no additional dying step;	may need auxiliary agents;
		suitable for thermally labile drugs;	long process time;
		simple to design.	low productivity (batch).
	SAS-GAS: a batch process where drug and cyclodextrin are first dissolved in a liquid solvent and then contacted with scCO_2_ that acts as an antisolvent causing the precipitation of the complexes.	reduced residual solvent;	complex to design;
		no additional dying step;	low productivity (batch).
		suitable for thermally labile drugs;	
		control of particle size;	
		no need of nozzles.	
	SAS-ASES/PCA: a semicontinuous process where a solution containing drug and cyclodextrin is injected into a precipitation vessel through an atomization nozzle. The vessel is also fed with scCO_2_ that acts as an antisolvent.	reduced residual solvent;	complex to design;
		no additional dying step;	possible nozzle blockage.
		suitable for thermally labile drugs;	
		good control of particle size.	
	
	SAS-SEDS: differs from ASES/PCA for the atomization device, a coaxial nozzle that provides simultaneous introduction of the solution and scCO_2_.	reduced residual solvent;	complex to design;
		no additional dying step;	possible nozzle blockage.
		suitable for thermally labile drugs;	
		best control of particle size.	
	
	SAS-ARISE: a batch process exploiting a pressure difference to achieve mixing between a solution containing drug and cyclodextrin, and scCO_2_ that acts as an antisolvent.	reduced residual solvent;	complex to design;
		no additional dying step;	low productivity (batch).
		suitable for thermally labile drugs;	
		control of particle size;	
		no need of nozzles.	
	SAA/SASD: scCO_2_ is dissolved in a solution containing drug and cyclodextrin, which is spray dried at atmospheric conditions.	reduced residual solvent;	complex to design;
		no additional dying step;	possible nozzle blockage.
		suitable for thermally labile drugs;	
		good control of particle size.	
Conventional technologies	Co-grinding: physical mixtures of drug and cyclodextrin are co-grinded in a ball mill.	simple to design;	high mechanical stress;
		no use of organic solvents;	high thermal stress;
		control of particle size.	low inclusion efficiency.
	Kneading: drug and cyclodextrin are mixed in presence of a solvent. After drying, the residual is pulverized.	simple to design.	organic solvent residues;
			low inclusion efficiency.
	
	Sealed heating: physical mixtures of drug and cyclodextrin are sealed in a container in the presence of small amounts of a solvent. The container is heated for a fixed period. After treatment, complex is desiccated to remove solvent traces.	solvent is generally water;	high thermal stress;
		simple to design.	desiccation step is required;
			no control of particle size.
	
	
	
	
	Spray drying: a solution containing drug and cyclodextrin is sprayed through a nozzle in a drying chamber.	good control of particle size;	possible nozzle blockage.
		good inclusion efficiency.	organic solvent residues;
			high thermal stress.
	
	Freeze drying: a solution containing drug and cyclodextrin is frozen and then lyophilized.	good inclusion efficiency.	organic solvent residues;
			long process times;
			no control of particle size.
	Co-evaporation: a solution containing drug and cyclodextrin is heated to remove solvents. The precipitate is desiccated to remove solvent traces.	good inclusion efficiency.	high thermal stress;
			organic solvent residues;
			long process times;
			no control of particle size;
			desiccation step is required.
	Coprecipitation: a solution containing drug and cyclodextrin is cooled to achieve complex precipitation. The precipitate is filtered, washed, and dried.	good inclusion efficiency.	organic solvent residues;
			long process times.
			drying step is required;
			no control of particle size.
	

## Data Availability

Not applicable.

## Abbreviations

ARISE	Atomized rapid injection solvent extraction
ASES	Aerosol solvent extraction system
DCM	Dichloromethane
DMF	Dimethylformamide
DMSO	Dimethyl sulfoxide
DMβCD	Dimethyl-β-cyclodextrin
DSC	Differential scanning calorimetry
FAγCD	Perfluorobutanoyl-γ-cyclodextrin
FTIR	Fourier transform infrared
GAS	Gas antisolvent
HPMC	Hydroxypropylmethyl cellulose
HPV	Hydroxypropyl cellulose
HPβCD	Hydroxypropyl-β-cyclodextrin
HPγCD	Hydroxypropyl-γ-cyclodextrin
MβCD	Methyl-β-cyclodextrin
NMR	Nuclear magnetic resonance
NSAID	Non-steroidal anti-inflammatory drug
PAβCD	Peracetylated-β-cyclodextrin
PCA	Precipitation with compressed antisolvent
PEG	Polyethylene glycol
PMMA	Ppoly methyl methacrylate
PVP	Polyvinyl pyrrolidone
PVP-VA	Polyvinyl pyrrolidone-vinyl acetate
RESS	Rapid expansion of supercritical solutions
SAA	Supercritical-assisted atomization
SAS	Supercritical antisolvent
SASD	Supercritical-assisted spray drying
scCO_2_	Supercritical carbon dioxide
SEDS	Solution-enhanced dispersion by supercritical fluids
SEM	Scanning electron microscopy
SFC	Supercritical fluid complexation
SSI	Supercritical solvent impregnation
TAβCD	Triacetyl-β-cyclodextrin
TMβCD	Trimethyl-β-cyclodextrin
αCD	α-Cyclodextrin
βCD	β-Cyclodextrin
γCD	γ-Cyclodextrin

## References

[1] Adeoye O., Cabral-Marques H. (2017). Cyclodextrin nanosystems in oral drug delivery: A mini review. Int. J. Pharm..

[2] Jansook P., Ogawa N., Loftsson T. (2018). Cyclodextrins: Structure, physicochemical properties and pharmaceutical applications. Int. J. Pharm..

[3] Carneiro S.B., Costa Duarte F.Í., Heimfarth L., Siqueira Quintans J.D., Quintans-Júnior L.J., Veiga Júnior V.F., Neves de Lima Á.A. (2019). Cyclodextrin–drug inclusion complexes: In vivo and in vitro approaches. Int. J. Mol. Sci..

[4] Knez Ž., Markočič E., Leitgeb M., Primožič M., Knez Hrnčič M., Škerget M. (2014). Industrial applications of supercritical fluids: A review. Energy.

[5] Manjare S.D., Dhingra K. (2019). Supercritical fluids in separation and purification: A review. Mater. Sci. Energy Technol..

[6] Liu G., Li J., Deng S. (2021). Applications of supercritical anti-solvent process in preparation of solid multicomponent systems. Pharmaceutics.

[7] Banchero M. (2020). Recent advances in supercritical fluid dyeing. Color. Technol..

[8] Kankala R.K., Zhang Y.S., Wang S.-B., Lee C.-H., Chen A.-Z. (2017). Supercritical fluid technology: An emphasis on drug delivery and related biomedical applications. Adv. Healthc. Mater..

[9] Padrela L., Rodrigues M.A., Duarte A., Dias A.M.A., Braga M.E.M., de Sousa H.C. (2018). Supercritical carbon dioxide-based technologies for the production of drug nanoparticles/nanocrystals–a comprehensive review. Adv. Drug Deliver. Rev..

[10] García-González C.A., Sosnik A., Kalmár J., De Marco I., Erkey C., Concheiro A., Alvarez-Lorenzo C. (2021). Aerogels in drug delivery: From design to application. J. Controll. Release.

[11] Lee S.-Y., Jung I.-I., Kim J.-K., Lim G.-B., Ryu J.-H. (2008). Preparation of itraconazole/HP-β-CD inclusion complexes using supercritical aerosol solvent extraction system and their dissolution characteristics. J. Supercrit. Fluid..

[12] Kamihira M., Asai T., Yamagata Y., Taniguchi M., Kobayashi T. (1990). Formation of inclusion complexes between cyclodextrins and aromatic compounds under pressurized carbon dioxide. J. Ferment. Bioeng..

[13] Mammucari R., Foster N.R. (2008). Dense gas technology and cyclodextrins: State of the art and potential. Curr. Org. Chem..

[14] Shehatta I., Al-Marzouqi A.H., Jobe B., Dowaidar A. (2005). Enhancement of aqueous solubility of itraconazole by complexation with cyclodextrins using supercritical carbon dioxide. Can. J. Chem..

[15] Van Hees T., Evrard B., Piel G., Delattre L. A Comparative study of the dissolution properties of piroxicam-β-cyclodextrin inclusion complexes prepared by different methods. Proceedings of the 9th Symposium on Cyclodextrins.

[16] Van Hees T., Piel G., Evrard B., Otte X., Thunus L., Delattre L. (1999). Application of supercritical carbon dioxide for the preparation of a piroxicam-β-cyclodextrin inclusion compound. Pharm. Res..

[17] Junco S., Casimiro T., Ribeiro N., Da Ponte M.N., Cabral Marques H.M. (2002). Optimisation of supercritical carbon dioxide systems for complexation of naproxen: Beta-cyclodextrin. J. Incl. Phenom. Macrocycl. Chem..

[18] Junco S., Casimiro T., Ribeiro N., Ponte M.N.D., Marques H.C. (2002). A comparative study of naproxen–beta cyclodextrin complexes prepared by conventional methods and using supercritical carbon dioxide. J. Incl. Phenom. Macrocycl. Chem..

[19] Van Hees T., Barillaro V., Piel G., Bertholet P., De Hassonville S., Evrard B., Delattre L. (2002). Application of supercritical carbon dioxide for the preparation of drug-cyclodextrin inclusion compounds. J. Incl. Phenom. Macrocycl. Chem..

[20] Locci E., Lai S., Piras A., Marongiu B., Lai A. (2004). ^13^ C-CPMAS and ^1^ H-NMR study of the inclusion complexes of β-cyclodextrin with carvacrol, thymol, and eugenol prepared in supercritical carbon dioxide. Chem. Biodivers..

[21] Moneghini M., Kikic I., Perissutti B., Franceschinis E., Cortesi A. (2004). Characterisation of nimesulide–betacyclodextrins systems prepared by supercritical fluid impregnation. Eur. J. Pharm. Biopharm..

[22] Barillaro V., Bertholet P., de Hassonville S.H. (2004). Effect of acidic ternary compounds on the formation of miconazole/cyclodextrin inclusion complexes by means of supercritical carbon dioxide. J. Pharm. Pharm. Sci..

[23] Barillaro V., Dive G., Bertholet P., Evrard B., Delattre L., Eric Z., Piel G. (2007). Theoretical and experimental investigations on miconazole/cyclodextrin/acid complexes: Molecular modeling studies. Int. J. Pharm..

[24] Al-Marzouqi A.H., Shehatta I., Jobe B., Dowaidar A. (2006). Phase solubility and inclusion complex of itraconazole with β-cyclodextrin using supercritical carbon dioxide. J. Pharm. Sci..

[25] Hassan H.A., Al-Marzouqi A.H., Jobe B., Hamza A.A., Ramadan G.A. (2007). Enhancement of dissolution amount and in vivo bioavailability of itraconazole by complexation with β-cyclodextrin using supercritical carbon dioxide. J. Pharmaceut. Biomed..

[26] Tozuka Y., Fujito T., Moribe K., Yamamoto K. (2006). Ibuprofen-cyclodextrin inclusion complex formation using supercritical carbon dioxide. J. Incl. Phenom. Macrocycl. Chem..

[27] Hussein K., Türk M., Wahl M.A. (2007). Comparative evaluation of ibuprofen/β-cyclodextrin complexes obtained by supercritical carbon dioxide and other conventional methods. Pharm. Res..

[28] Türk M., Upper G., Steurenthaler M., Hussein K., Wahl M.A. (2007). Complex formation of ibuprofen and β-cyclodextrin by controlled particle deposition (CPD) using SC-CO_2_. J. Supercrit. Fluid..

[29] Hussein K., Türk M., Wahl M.A. (2008). Drug loading into β-cyclodextrin granules using a supercritical fluid process for improved drug dissolution. Eur. J. Pharm. Sci..

[30] Al-Marzouqi A.H., Jobe B., Dowaidar A., Maestrelli F., Mura P. (2007). Evaluation of supercritical fluid technology as preparative technique of benzocaine–cyclodextrin complexes—Comparison with conventional methods. J. Pharmaceut. Biomed..

[31] Al-Marzouqi A., Jobe B., Corti G., Cirri M., Mura P. (2007). Physicochemical characterization of drug-cyclodextrin complexes prepared by supercritical carbon dioxide and by conventional techniques. J. Incl. Phenom. Macrocycl. Chem..

[32] Bounaceur A., Rodier E., Fages J. (2007). Maturation of a ketoprofen/β-cyclodextrin mixture with supercritical carbon dioxide. J. Supercrit. Fluid..

[33] Al-Marzouqi A.H., Solieman A., Shehadi I., Adem A. (2008). Influence of the preparation method on the physicochemical properties of econazole-β-cyclodextrin complexes. J. Incl. Phenom. Macrocycl. Chem..

[34] Sauceau M., Rodier E., Fages J. (2008). Preparation of inclusion complex of piroxicam with cyclodextrin by using supercritical carbon dioxide. J. Supercrit. Fluid..

[35] Barillaro V., Dive G., Bertholet P., Evrard B., Delattre L., Frederich M., Ziémons E., Piel G. (2008). Theoretical and experimental investigations of organic acids/cyclodextrin complexes and their consequences upon the formation of miconazole/cyclodextrin/acid ternary inclusion complexes. Int. J. Pharm..

[36] Al-Marzouqi A.H., Elwy H.M., Shehadi I., Adem A. (2009). Physicochemical properties of antifungal drug–cyclodextrin complexes prepared by supercritical carbon dioxide and by conventional techniques. J. Pharmaceut. Biomed..

[37] Grandelli H.E., Hassler J.C., Whittington A., Kiran E. (2012). Melting point depression of piroxicam in carbon dioxide+co-solvent mixtures and inclusion complex formation with β-cyclodextrin. J. Supercrit. Fluid..

[38] Banchero M., Manna L. (2012). The use of lysine to enhance the SFC of ketoprofen and cyclodextrins. J. Supercrit. Fluid..

[39] Rahim R., Trisanti P.N. (2017). Ketoprofen-β−cyclodextrin inclusion complexes formation by supercritical process. AIP Conf. Proc..

[40] Valarini Junior O., Dantas J.H., Barão C.E., Zanoelo E.F., Cardozo-Filho L., de Moraes F.F. (2017). Formation of inclusion compounds of (+)catechin with β-cyclodextrin in different complexation media: Spectral, thermal and antioxidant properties. J. Supercrit. Fluid..

[41] Abbaszadegan S., Al-Marzouqi A.H., Salem A.A., Amin A. (2015). Physicochemical characterizations of safranal-β-cyclodextrin inclusion complexes prepared by supercritical carbon dioxide and conventional methods. J. Incl. Phenom. Macrocycl. Chem..

[42] Goenawan J., Trisanti P.N. (2015). Sumarno the influence of dissolved H_2_ O content in supercritical carbon dioxide to the inclusion complexes formation of ketoprofen/β-cyclodextrin. AIP Conf. Proc..

[43] Trisanti P.N. (2019). Sumarno the effect of water addition in inclusion formation of ketoprofen/β-cyclodextrin using supercritical CO_2_. AIP Conf. Proc..

[44] Lei H.P., Zhang H., Ge F.H., Ye Z.W., Li C.H. (2018). Preparation of a menthol/β-cyclodextrin inclusion complex using supercritical CO_2_. Indian J. Pharm. Sci..

[45] Al-Shar’i N.A., Obaidat R.M. (2018). Experimental and computational comparative study of the supercritical fluid technology (SFT) and kneading method in preparing β-cyclodextrin complexes with two essential oils (Linalool and Carvacrol). AAPS PharmSciTech.

[46] Lee C.-W., Kim S.-J., Youn Y.-S., Widjojokusumo E., Lee Y.-H., Kim J., Lee Y.-W., Tjandrawinata R.R. (2010). Preparation of bitter taste masked cetirizine dihydrochloride/β-cyclodextrin inclusion complex by supercritical antisolvent (SAS) process. J. Supercrit. Fluid..

[47] Nerome H., Machmudah S., Fukuzato R., Higashiura T., Youn Y.-S., Lee Y.-W., Goto M. (2013). Nanoparticle formation of lycopene/β-cyclodextrin inclusion complex using supercritical antisolvent precipitation. J. Supercrit. Fluid..

[48] Lei H.P., Zhang K.R., Wang J., Zhang H., Shi Q.L., Ge F.H., Han Q.B. (2019). Nanoparticle formation of puerarin-beta-cyclodextrin inclusion complex using SEDS: Dissolution enhancement. Indian J. Pharm. Sci..

[49] Jia J., Zhang K., Zhou X., Zhou D., Ge F. (2018). Precise dissolution control and bioavailability evaluation for insoluble drug berberine via a polymeric particle prepared using supercritical CO_2_. Polymers.

[50] Franco P., De Marco I. (2021). Preparation of non-steroidal anti-inflammatory drug/β-cyclodextrin inclusion complexes by supercritical antisolvent process. J. CO2 Util..

[51] Rosas M.D., Piqueras C.M., Piva G.K., Ramírez-Rigo M.V., Filho L.C., Bucalá V. (2021). Simultaneous formation of inclusion complex and microparticles containing albendazole and β-cyclodextrin by supercritical antisolvent co-precipitation. J. CO2 Util..

[52] Rodier E., Lochard H., Sauceau M., Letourneau J.-J., Freiss B., Fages J. (2005). A three step supercritical process to improve the dissolution rate of eflucimibe. Eur. J. Pharm. Sci..

[53] Ndayishimiye J., Popat A., Kumeria T., Blaskovich M.A.T., Robert Falconer J. (2021). Supercritical carbon dioxide assisted complexation of benznidazole: γ-cyclodextrin for improved dissolution. Int. J. Pharm..

[54] Toropainen T., Velaga S., Heikkilä T., Matilainen L., Jarho P., Carlfors J., Lehto V., Järvinen T., Järvinen K. (2006). Preparation of budesonide/γ-cyclodextrin complexes in supercritical fluids with a novel SEDS method. J. Pharm. Sci..

[55] Toropainen T., Heikkilä T., Leppänen J., Matilainen L., Velaga S., Jarho P., Carlfors J., Lehto V.-P., Järvinen T., Järvinen K. (2007). Crystal structure changes of γ-cyclodextrin after the SEDS process in supercritical carbon dioxide affect the dissolution rate of complexed budesonide. Pharm. Res..

[56] Shikhar A., Bommana M.M., Gupta S.S., Squillante E. (2011). Formulation development of carbamazepine–nicotinamide co-crystals complexed with γ-cyclodextrin using supercritical fluid process. J. Supercrit. Fluid..

[57] Adeoye O., Conceição J., Serra P.A., Bento da Silva A., Duarte N., Guedes R.C., Corvo M.C., Aguiar-Ricardo A., Jicsinszky L., Casimiro T. (2020). Cyclodextrin solubilization and complexation of antiretroviral drug lopinavir: In silico prediction; effects of derivatization, molar ratio and preparation method. Carbohyd. Polym..

[58] Bandi N., Wei W., Roberts C.B., Kotra L.P., Kompella U.B. (2004). Preparation of budesonide– and indomethacin–hydroxypropyl-β-cyclodextrin (HPBCD) complexes using a single-step, organic-solvent-free supercritical fluid process. Eur. J. Pharm. Sci..

[59] He J. (2009). Complex of Shikonin and β-cyclodextrins by using supercritical carbon dioxide. J. Incl. Phenom. Macrocycl. Chem..

[60] Temtem M., Pompeu D., Jaraquemada G., Cabrita E.J., Casimiro T., Aguiar-Ricardo A. (2009). Development of PMMA membranes functionalized with hydroxypropyl-β-cyclodextrins for controlled drug delivery using a supercritical CO_2_-assisted technology. Int. J. Pharm..

[61] He J., Li W. (2009). Preparation of borneol–methyl-β-cyclodextrin inclusion complex by supercritical carbon dioxide processing. J. Incl. Phenom. Macrocycl. Chem..

[62] He J. (2010). Complex between modified β-cyclodextrins and three components of traditional Chinese medicine in supercritical carbon dioxide medium. J. Incl. Phenom. Macrocycl. Chem..

[63] Banchero M., Manna L. (2011). Investigation of the piroxicam/hydroxypropyl-β-cyclodextrin inclusion complexation by means of a supercritical solvent in the presence of auxiliary agents. J. Supercrit. Fluid..

[64] Pan H., Wang H.-B., Yu Y.-B., Cheng B.-C., Wang X.-Y., Li Y. (2017). A superior preparation method for daidzein-hydroxypropyl-β-cyclodextrin complexes with improved solubility and dissolution: Supercritical fluid process. Acta Pharm..

[65] Wang H.-B., Yang F.-F., Gai X.-M., Cheng B.-C., Li J.-Y., Pan H., Yang X.-G., Pan W.-S. (2017). A PH-independent instantaneous release of flurbiprofen: A study of the preparation of complexes, their characterization and in vitro/in vivo evaluation. Drug Dev. Ind. Pharm..

[66] Li Y., He Z.-D., Zheng Q.-E., Hu C., Lai W.-F. (2018). Hydroxypropyl-β-cyclodextrin for delivery of baicalin via inclusion complexation by supercritical fluid encapsulation. Molecules.

[67] Pires F.Q., Pinho L.A., Freire D.O., Silva I.C.R., Sa-Barreto L.L., Cardozo-Filho L., Gratieri T., Gelfuso G.M., Cunha-Filho M. (2019). Thermal analysis used to guide the production of thymol and lippia origanoides essential oil inclusion complexes with cyclodextrin. J. Therm. Anal. Calorim..

[68] Mammucari R., Dehghani F., Foster N.R. (2006). Dense gas processing of micron-sized drug formulations incorporating hydroxypropylated and methylated beta-cyclodextrin. Pharm. Res..

[69] Jun S.W., Kim M.-S., Kim J.-S., Park H.J., Lee S., Woo J.-S., Hwang S.-J. (2007). Preparation and characterization of simvastatin/hydroxypropyl-β-cyclodextrin inclusion complex using supercritical antisolvent (SAS) process. Eur. J. Pharm. Biopharm..

[70] Zhou R., Wang F., Guo Z., Zhao Y.L. (2012). Peparation and characterization of resveratrol/hydroxypropyl-β-cyclodextrin inclusion complex using supercritical antisolvent technology. J. Food Process Eng..

[71] Kim M.S. (2013). Influence of hydrophilic additives on the supersaturation and bioavailability of dutasteride-loaded hydroxypropyl-β-cyclodextrin nanostructures. Int. J. Nanomed..

[72] Huang Y., Zu Y., Zhao X., Wu M., Feng Z., Deng Y., Zu C., Wang L. (2016). Preparation of inclusion complex of apigenin-hydroxypropyl-β-cyclodextrin by using supercritical antisolvent process for dissolution and bioavailability enhancement. Int. J. Pharm..

[73] Kurniawansyah F., Duong H.T.T., Luu T.D., Mammucari R., Vittorio O., Boyer C., Foster N. (2015). Inhalable curcumin formulations: Micronization and bioassay. Chem. Eng. J..

[74] Kurniawansyah F., Mammucari R., Foster N.R. (2015). Inhalable curcumin formulations by supercritical technology. Powder Technol..

[75] Bommana M.M., Kirthivasan B., Shikhar A., Gupta S.S., Squillante E. (2014). In vivo brain microdialysis as a formulation-screening tool for a poorly soluble centrally acting drug. Drug Dev. Ind. Pharm..

[76] Yan T., Zhang Y., Ji M., Wang Z., Yan T. (2019). Preparation of irbesartan composite microparticles by supercritical aerosol solvent extraction system for dissolution enhancement. J. Supercrit. Fluid..

[77] Di Capua A., Bejarano A., Adami R., Reverchon E. (2018). Preparation and characterization of chilean propolis coprecipitates using supercritical assisted atomization. Chem. Eng. Res. Des..

[78] Sun J., Hong H., Zhu N., Han L., Suo Q. (2019). Response surface methodology to optimize the preparation of tosufloxacin tosylate/hydroxypropyl-β-cyclodextrin inclusion complex by supercritical antisolvent process. J. Mol. Struct..

[79] Sun J., Hong H., Zhu N., Han L., Suo Q. (2020). Spectroscopic analysis and dissolution properties study of tosufloxacin tosylate/hydroxypropyl-β-cyclodextrin inclusion complex prepared by solution-enhanced dispersion with supercritical CO_2_. J. Pharm. Innov..

[80] Yan T., Ji M., Sun Y., Yan T., Zhao J., Zhang H., Wang Z. (2020). Preparation and characterization of baicalein/hydroxypropyl-β-cyclodextrin inclusion complex for enhancement of solubility, antioxidant activity and antibacterial activity using supercritical antisolvent technology. J. Incl. Phenom. Macrocycl. Chem..

[81] Barillaro V., Evrard B., Delattre L., Piel G. (2005). Oral bioavailability in pigs of a miconazole/hydroxypropyl-γ-cyclodextrin/l-tataric acid inclusion complex produced by supercritical carbon dioxide processing. AAPS J..

[82] Barillaro V., Dive G., Ziémons E., Bertholet P., Evrard B., Delattre L., Piel G. (2008). Theoretical and experimental vibrational study of miconazole and its dimers with organic acids: Application to the IR characterization of its inclusion complexes with cyclodextrins. Int. J. Pharm..

[83] Adeoye O., Costa C., Casimiro T., Aguiar-Ricardo A., Cabral-Marques H. (2018). Preparation of ibuprofen/hydroxypropyl-γ-cyclodextrin inclusion complexes using supercritical CO_2_-assisted spray drying. J. Supercrit. Fluid..

[84] Charoenchaitrakool M., Dehghani F., Foster N.R. (2002). Utilization of supercritical carbon dioxide for complex formation of ibuprofen and methyl-β-cyclodextrin. Int. J. Pharm..

[85] He J., Li W. (2009). Complex formation of cinnamaldehyde-methyl-β-cyclodextrin and muscone-methyl-β-cyclodextrin by supercritical carbon dioxide processing and sealed heating method. J. Incl. Phenom. Macrocycl. Chem..

[86] Banchero M., Ronchetti S., Manna L. (2013). characterization of ketoprofen/methyl- *β* -cyclodextrin complexes prepared using supercritical carbon dioxide. J. Chem..

[87] Rudrangi S.R.S., Kaialy W., Ghori M.U., Trivedi V., Snowden M.J., Alexander B.D. (2016). Solid-state flurbiprofen and methyl-β-cyclodextrin inclusion complexes prepared using a single-step, organic solvent-free supercritical fluid process. Eur. J. Pharm. Biopharm..

[88] Rudrangi S.R.S., Trivedi V., Mitchell J.C., Wicks S.R., Alexander B.D. (2015). Preparation of olanzapine and methyl-β-cyclodextrin complexes using a single-step, organic solvent-free supercritical fluid process: An approach to enhance the solubility and dissolution properties. Int. J. Pharm..

[89] Rudrangi S.R.S., Bhomia R., Trivedi V., Vine G.J., Mitchell J.C., Alexander B.D., Wicks S.R. (2015). Influence of the preparation method on the physicochemical properties of indomethacin and methyl-β-cyclodextrin complexes. Int. J. Pharm..

[90] Moribe K., Fujito T., Tozuka Y., Yamamoto K. (2007). Solubility-dependent complexation of active pharmaceutical ingredients with trimethyl-β-cyclodextrin under supercritical fluid condition. J. Incl. Phenom. Macrocycl. Chem..

[91] Ganapathy H.S., Lee M.Y., Park C., Lim K.T. (2008). Sustained release applications of a fluoroalkyl ester-functionalized amphiphilic cyclodextrin by inclusion complex formation with water-soluble drugs in supercritical carbon dioxide. J. Fluor. Chem..

[92] Lee M.Y., Ganapathy H.S., Lim K.T. (2010). Controlled drug release applications of the inclusion complex of peracetylated-β-cyclodextrin and water-soluble drugs formed in supercritical carbon dioxide. J. Phys. Chem. Solids.

[93] Ganapathy H.S., Woo M.H., Gal Y.S., Lim K.T. (2007). Inclusion complex formation of water- soluble drug, captopril, and peracetylated-β-cyclodextrin in supercritical co_2_ for controlled release applications. Key Eng. Mat..

[94] Sultana T., Jung J.M., Hong S.-S., Lee W.-K., Gal Y.-S., Kim H.G., Lim K.T. (2012). Characteristic profiles of the inclusion complex of omeprazole/peracylated-β-cyclodextrin formed in supercritical carbon dioxide. J. Incl. Phenom. Macrocycl. Chem..

[95] Ingrosso F., Altarsha M., Dumarçay F., Kevern G., Barth D., Marsura A., Ruiz-López M.F. (2016). Driving forces controlling host-guest recognition in supercritical carbon dioxide solvent. Chem. Eur. J..

[96] Maia-Obi L.P., Vidinha P., Ferraz H.G., Bazito R.C. (2021). Non-inclusion complexation of peracetylated β-cyclodextrin with ibuprofen in supercritical carbon dioxide. J. Supercrit. Fluids.

[97] Ivanova G.I., Vão E.R., Temtem M., Aguiar-Ricardo A., Casimiro T., Cabrita E.J. (2009). High-Pressure NMR characterization of triacetyl-β-cyclodextrin in supercritical carbon dioxide: HP-NMR of triacetyl-β-cyclodextrin in ScCO_2_. Magn. Reson. Chem..

[98] Nunes A.V.M., Almeida A.P.C., Marques S.R., de Sousa A.R.S., Casimiro T., Duarte C.M.M. (2010). Processing triacetyl-β-cyclodextrin in the liquid phase using supercritical CO_2_. J. Supercrit. Fluid..

[99] Brewster M.E., Loftsson T. (2007). Cyclodextrins as pharmaceutical solubilizers. Adv. Drug Deliv. Rev..

[100] Mura P. (2014). Analytical techniques for characterization of cyclodextrin complexes in aqueous solution: A review. J. Pharmaceut. Biomed..

[101] Mura P. (2015). Analytical techniques for characterization of cyclodextrin complexes in the solid state: A review. J. Pharmaceut. Biomed..

[102] Altarsha M., Ingrosso F., Ruiz-López M.F. (2012). Cavity closure dynamics of peracetylated β-cyclodextrins in supercritical carbon dioxide. J. Phys. Chem. B.

[103] Türk M., Kraska T. (2009). Experimental and theoretical investigation of the phase behavior of naproxen in supercritical CO_2_. J. Chem. Eng. Data.

[104] Manna L., Banchero M. (2018). Solubility of tolbutamide and chlorpropamide in supercritical carbon dioxide. J. Chem. Eng. Data.

[105] Franco P., De Marco I. (2021). Nanoparticles and nanocrystals by supercritical CO_2_-assisted techniques for pharmaceutical applications: A review. Appl. Sci..

[106] Prosapio V., De Marco I., Reverchon E. (2018). Supercritical antisolvent coprecipitation mechanisms. J. Supercrit. Fluids.

[107] Reverchon E., Antonacci A. (2006). Cyclodextrins micrometric powders obtained by supercritical fluid processing. Biotechnol. Bioeng..

[108] Franco P., De Marco I. (2020). Supercritical antisolvent process for pharmaceutical applications: A review. Processes.

[109] He Y., Hou X., Guo J., He Z., Guo T., Liu Y., Zhang Y., Zhang J., Feng N. (2020). Activation of a gamma–cyclodextrin–based metal–organic framework using supercritical carbon dioxide for high–efficient delivery of honokiol. Carbohyd. Polym..

